# MetaZooGene Intercalibration Experiment (MZG‐ICE): Metabarcoding Marine Zooplankton Diversity of the Global Ocean

**DOI:** 10.1111/1755-0998.70090

**Published:** 2025-12-26

**Authors:** Leocadio Blanco‐Bercial, Jennifer M. Questel, Paola G. Batta‐Lona, Ruben Escribano, Tone Falkenhaug, Junya Hirai, Jenny A. Huggett, Pedro Martinez Arbizu, Katja T. C. A. Peijnenburg, Leonie Suter, Agata Weydmann‐Zwolicka, Stacey Dubbeldam, Elza Duijm, Elizaveta Ershova‐Menze, Carolina E. Gonzalez, Ashrenee Govender, Johan Groeneveld, Sahar Khodami, Anna J. MacDonald, Monika Mioduchowska, Andrea M. Polanowski, Rocio Rodriguez‐Perez, Todd D. O'Brien, Ann Bucklin

**Affiliations:** ^1^ Bermuda Institute of Ocean Sciences Arizona State University St. Georges Bermuda; ^2^ Institute of Marine Science University of Alaska Fairbanks Fairbanks Alaska USA; ^3^ Department of Marine Sciences University of Connecticut – Avery Point Groton Connecticut USA; ^4^ Instituto Milenio de Oceanografía Universidad de Concepción Concepción Chile; ^5^ Flødevigen Research Station Institute of Marine Research Arendal Norway; ^6^ Atmosphere and Ocean Research Institute The University of Tokyo Chiba Japan; ^7^ Department of Forestry, Fisheries and the Environment Oceans and Coastal Research Cape Town South Africa; ^8^ Senckenberg Am Meer, DZMB Wilhelmshaven Niedersachsen Germany; ^9^ Naturalis Biodiversity Center Leiden the Netherlands; ^10^ Institute for Biodiversity and Ecosystem Dynamics (IBED) University of Amsterdam Amsterdam the Netherlands; ^11^ Australian Antarctic Division Department of Climate Change, Energy, the Environment and Water Kingston Tasmania Australia; ^12^ Faculty of Oceanography and Geography, Department of Marine Biology and Biotechnology University of Gdańsk Gdynia Poland; ^13^ Oceanographic Research Institute Durban KwaZulu‐Natal South Africa; ^14^ Faculty of Biology, Department of Evolutionary Genetics and Biosystematics University of Gdańsk Gdańsk Poland; ^15^ NOAA National Marine Fisheries Service Silver Spring Maryland USA

**Keywords:** biodiversity, DNA metabarcoding, ecosystem monitoring, intercalibration, marine zooplankton

## Abstract

DNA metabarcoding of zooplankton biodiversity is used increasingly for monitoring global ocean ecosystems, requiring comparable data from different research laboratories and ocean regions. The MetaZooGene Intercalibration Experiment (MZG‐ICE) was designed to examine1 and analyse patterns of variation of DNA sequence data resulting from multi‐gene metabarcoding of 10 zooplankton samples carried out by 10 research groups affiliated with the Scientific Committee for Ocean Research (SCOR). Aliquots of DNA extracted from the 10 zooplankton samples were distributed to MZG‐ICE groups for metabarcoding of four gene regions: V1‐V2, V4 and V9 of nuclear 18S rRNA and mitochondrial COI. Molecular protocols and procedures were recommended; substitutions were allowed as necessary. Resulting data were uploaded to a common repository for centralised statistics and bioinformatics. Based on proportional sequence numbers for abundant phyla, overall patterns of variation were consistent across many—but not all—MZG‐ICE groups. V9 showed highest similarity, followed (in order) by V4, V1‐V2, and COI. Outlier data were hypothesised to result from the use of different PCR protocols and sequencing platforms, and possible contamination. MZG‐ICE results indicated that DNA metabarcoding data from different laboratories and research groups can provide reliable, accurate and valid descriptions of biodiversity of zooplankton throughout the ocean. Recommendations included: pre‐screening QA/QC of raw data, detailed records for laboratory protocols, reagents, and instrumentation, and centralised bioinformatics and multivariate statistics. In the absence of universal agreement on standardised protocols or best practices, intercalibration is the best way forward toward validation of DNA metabarcoding of zooplankton diversity for global ocean monitoring.

## 1 Introduction

Molecular approaches are used with increasing frequency to characterise the diversity and abundance of marine organisms for research, monitoring, and management of ocean ecosystems (Goodwin et al. 2019; Rogers et al. 2022). DNA metabarcoding, entailing high‐throughput sequencing of environmental samples (Taberlet et al. 2012), is yielding new insights into biodiversity of marine ecosystems (Yebra et al. 2022; Bush et al. 2023), impacts of climate change, commercial fishing and other human activities (Andujar et al. 2018; Macher et al. 2025). DNA metabarcoding is a well‐established tool for analysis of diversity of marine zooplankton (Mohrbeck et al. 2015; Hirai et al. 2015, 2021; Deagle et al. 2018; Singh et al. 2021; Gonzalez et al. 2023; Kim et al. 2025). Depending upon the gene regions selected, metabarcoding can enable discrimination and accurate identification of cryptic species and detection of rare and invasive species, as well as meroplanktonic larvae of benthic invertebrates (Leray and Knowlton 2017; Schroeder et al. 2021).

Metabarcoding of DNA extracted from samples collected during time‐series ecosystem monitoring programs is being integrated into fisheries management and assessment programs in many ocean regions, including the Northwest Atlantic (Bucklin et al. 2019, 2022; Blanco‐Bercial 2020), Northeast Pacific (Matthews et al. 2021; Questel et al. 2021), Mediterranean Sea (Di Capua et al. 2024), Australia (Deagle et al. 2018), South Atlantic (Huggett et al. 2022) and Southwest Indian Ocean (Govender et al. 2023). Analysis of DNA extracted from zooplankton samples uses many of the same methods as environmental DNA (eDNA), for which DNA is collected by filtration of seawater (Djurhuus et al. 2018; Suter et al. 2021; Gold et al. 2022; Sildever et al. 2023; Thompson and Thielen 2023; Klymus et al. 2024; Yang et al. 2024). The use of DNA metabarcoding as a foundation for management and conservation decision‐making throughout the global ocean will require documented evidence of accuracy, reliability and reproducibility of data and results (Wilding et al. 2023).

DNA metabarcoding entails a complex series of analytical steps, including DNA extraction, PCR amplification, library preparation, DNA sequencing, data quality control, bioinformatics and statistics. The methods used for DNA metabarcoding vary across the many research laboratories and commercial facilities that are responsible for analysis of samples from monitoring and assessment programs and projects. A number of studies have examined the impacts of the variety of reagents, protocols and procedures used for each step in the metabarcoding workflow from samples to data to conclusions (Alberdi et al. 2018; Jeunen et al. 2019; Zaiko et al. 2022; Govender et al. 2022; De Brauwer 2023; Ershova‐Menze et al. 2025; Vasselon et al. 2025).

Studies evaluated the accuracy and reliability of DNA metabarcoding of marine biodiversity based on comparative statistics and bioinformatics of results from different research laboratories (Clarke et al. 2017; Nagai et al. 2022; Ershova 2023; Hajibabaei 2022; Takahashi et al. 2023; Van den Bulcke et al. 2023; Doorenspleet et al. 2025). A study by Zaiko et al. (2022) examined metabarcoding data for marine biofouling communities from four research groups using different laboratory procedures, analytical workflows, and bioinformatics pipelines, and yielded recommendations to clearly articulate methods in publications and remove samples with low sequence numbers and evidence of contamination. Borja et al. (2024) used molecular indices of reference conditions for DNA metabarcoding of benthic marine ecosystems and recommended comparison of genomic and morphological methods to detect errors.

Intercalibration experiments are designed to evaluate the reliability of an analytical approach through open sharing of methodological details and intercomparison of results among multiple participating laboratories and facilities (Cutter 2013). A benchmark study by Berube et al. (2022) summarised and compared results from laboratories using various protocols and procedures for molecular analysis of marine microbial diversity resulting from a carefully designed intercalibration experiment. The report (Berube et al. 2022) also cited previous methodological intercomparisons for measurement of dissolved organic carbon (Sharp et al. 2002), dissolved inorganic carbon (Dickson et al. 2003), macronutrients (Becker et al. 2020) and trace metals (Schlitzer et al. 2018). Intercalibration can assess whether data from multiple sources should be included in public databases and can be considered a first step toward standardisation (Cutter 2013), although eventual selection of best practices requires controlled and replicated experiments testing protocols and procedures (Przeslawski et al. 2023). Efforts toward the goal of standardisation of marine biodiversity assessments have been carried out through the Ocean Best Practices (OBP) program (Pearlman et al. 2019; Samuel et al. 2021).

The MetaZooGene Intercalibration Experiment (MZG‐ICE) was designed to analyse and evaluate the patterns of variation of multi‐gene metabarcoding data produced by 10 participating research laboratories, starting with shared aliquots of DNA extracted from zooplankton samples collected by each group in different ocean regions. The data were uploaded and shared for centralised statistics and bioinformatics analysis. The focus of MZG‐ICE was on evaluating patterns of variation in descriptions of zooplankton diversity determined by different research laboratories carrying out analytical steps from DNA to data. The underlying strategy was based on the premise that standardisation of molecular protocols and procedures associated with DNA metabarcoding is unlikely—and perhaps impossible—across the many research laboratories and government agencies involved. In broad view, MZG‐ICE results can be used to evaluate the accuracy, reliability and reproducibility of DNA metabarcoding as a tool for ecosystem monitoring and conservation decision‐making throughout the global ocean.

## 2 Material and Methods

### 2.1 Selection and Preparation of Samples

**FIGURE 1:**
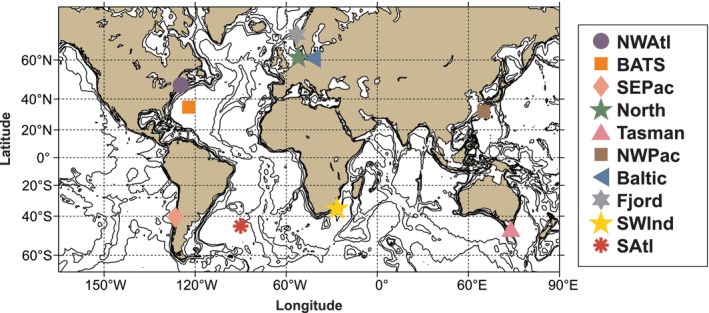
Map showing locations of the samples analysed in this manuscript. See Table 1 for explanation of sample name abbreviations and location coordinates for the 10 samples.

This study was designed by members of the MetaZooGene Working Group (WG157) of the Scientific Committee for Oceanic Research (SCOR). Each MZG‐ICE research group selected one zooplankton sample for analysis by all participating groups as part of the intercalibration experiment. Samples were preserved in ethanol (undenatured 70% or 95% ethyl alcohol) immediately upon collection and stored under refrigeration (0° to −20°C) prior to analysis. Samples provided by the 10 MZG‐ICE research groups were collected from different ocean regions and assigned descriptive names and abbreviations: Baltic Sea (Baltic), Bermuda Atlantic Time‐series Study (BATS), Southwest Indian Ocean (SWInd), North Sea (North), Northwest Atlantic (NWAtl), Northwest Pacific (NWPac), Northeast Atlantic Norwegian Fjord (Fjord), South Atlantic (SAtl), Southeast Pacific (SEPac) and Tasman Sea (Tasman) (Figure 1, Table 1).

**TABLE 1:** Sample designations, collection date and locations and full descriptive name of sample.

Sample	Collection date	Latitude/Longitude	Sample name
NWAtl	28‐Aug‐2019	43.0817 N, −69.5017 W	Northwest Atlantic Ocean
BATS	12‐May‐2021	31.6102 N, −64.2171 W	Bermuda Atlantic Time Series
SEPac	22‐Oct‐2021	−36.5222 S, −73.1375 W	Southeast Pacific Ocean
North	26‐Jul‐2022	54.5407 N, 8.7442 E	North Sea
Tasman	3‐Sep‐2018	−43.711 S, 147.977 E	Tasman Sea, South Pacific Ocean
NWPac	9‐Mar‐2022	29.173 N, 130.007 E	Northwest Pacific Ocean
Baltic	17‐May‐2022	54.6667 N, 19.1500 E	Baltic Sea
Fjord	5‐Nov‐2021	62.7082 N, 6.9872 E	Norwgian Fjord, Northeast Atlantic Ocean
SWInd	25‐May‐2022	−29.947 S, 31.099 E	Southwest Indian Ocean
SAtl	26‐Oct‐2017	−41.1433 S, −29.9945 W	South Atlantic Ocean

Each MZG‐ICE research group extracted and purified DNA from their selected sample using protocols and procedures, including reagents and kits, routinely used in their laboratory for metabarcoding analysis. Details for all protocols are summarised in Appendix S1. The DNA yield was recorded before division of each extract into 12 aliquots of equal concentration. Aliquots were either dried at room temperature (e.g., SpeedVac) or freeze‐dried (lyophilised). Each group shipped nine aliquots to the University of Connecticut (UConn) and retained three aliquots for their own analysis. At UConn, samples were organised, allocated, packaged and shipped under refrigeration to each MZG‐ICE laboratory, with appropriate customs labelling and permitting for each country.

### 2.2 PCR Amplification, Library Preparation, and DNA Sequencing

Gene regions for DNA metabarcoding were selected to allow analysis of zooplankton diversity at differing levels of taxonomic resolution and detection, including V1‐V2, V4, and V9 regions of nuclear 18S rRNA and mitochondrial cytochrome oxidase I (COI). Metabarcoding analysis was carried out for 10 samples for all four gene regions by each MZG‐ICE group following their own standard protocols and procedures for the many steps in the DNA metabarcoding analytical pipeline, including PCR primers and protocols, library preparation, and sequencing platforms and parameters (depth, length and directions). Some protocol methodologies, including PCR primer sequences, were recommended based on consensus agreement among the research groups (Table 2). However, groups were not discouraged from modifying PCR primers and protocols, reagents, adaptors and other reaction specifics routinely used in their own laboratory. Several groups used different PCR primers after obtaining poor results with those recommended (Table 2, Appendix S1). This approach was necessary to yield sufficient concentration and high‐quality amplification products from all research groups. Group #8 replaced the COI forward primer, mtCOIintF (Leray et al. 2013) with mlCOIintF‐XT (Wangensteen et al. 2018). Groups #2 and #9 amplified COI using the reverse primer HCO‐2198 (Folmer et al. 1994), instead of jgHCO‐2198 (Geller et al. 2013). These necessary replacements were expected, since the jgHCO2198 primer contains inosine, which is not compatible with the PCR polymerase enzymes used by those groups. Group #10 solved this problem by switching to Amplitaq Gold PCR mix (Applied Biosystems) for COI, which is compatible with inosine (Moretti et al. 1998). Another finding was the need for higher DNA concentration for successful amplification of COI, as shown by Group #10. Protocol details are provided Appendix S1.

**TABLE 2:** Recommended PCR primer sequences for each gene region.

Gene region	Primer name	Primer sequence	References
mtCOI	mlCOIintF	5′‐GGWACWGGWTGAACWGTWTAYCCYCC‐3′	Leray et al. (2013)
mtCOI	jgHCO2198	5′‐TAIACYTCIGGRTGICCRAARAAYCA‐3′	Geller et al. (2013)
V9 18S	1389F	5′‐TTGTACACACCGCCC‐3′	Amaral‐Zettler et al. (2009)
V9 18S	1510R	5′‐CCTTCYGCAGGTTCACCTAC‐3′	Amaral‐Zettler et al. (2009)
V1‐V2 18S	SSU_FO4	5′‐GCTTGTCTCAAAGATTAAGCC‐3′	Fonseca et al. (2010)
V1‐V2 18S	SSU_R22	5′‐GCCTGCTGCCTTCCTTGGA‐3′	Fonseca et al. (2010)
V4 18S	TAReuk454‐FWD1	5′‐CCAGCASCYGCGGTAATTCC‐3′	Stoeck et al. (2010)
V4 18S	TAReuk‐REV3	5′‐ACTTTCGTTCTTGATYRA‐3′	Stoeck et al. (2010)

MZG‐ICE groups were requested to carry out two replicate PCRs from their own samples and two additional replicates of their choosing. Not all groups provided results for replicates. The results from available replicate reactions were analysed and statistically compared. The results reported include only one of each of the replicates, which was chosen at random.

Library preparation protocols and procedures differed between MZG‐ICE groups, with some groups using Illumina kits and others preparing home‐made primers and links. Group #8 attached sequencing adapters via a PCR‐free approach for COI (NEXT‐Flex DNA Free Library preparation kit) and Group #10 used the Illumina Nextera XT DNA Library Preparation kit. In some cases, library preparation and sequencing steps were carried out in the research group's laboratory, while in others these procedures were done at a sequencing facility. Nine MZG‐ICE groups used Illumina MiSeq sequencing platforms; Group #3 used Illumina NextSeq (Appendix S1).

Pre‐treatment of data from two MZG‐ICE groups used Trimmomatic (Bolger et al. 2014). For V9, the methods used by the groups prevented the recovery of samples through Quality Control (QC). One group used 150 PE, which was too short to recover the full fragment in R1 and R2, preventing the use of a trim to overlap in Mothur (Schloss et al. 2009). Several groups used 300 PE chemistry, which caused a long tail of low‐quality sequence after ~175 base pairs (bp). This issue was resolved by using the CROP tool from Trimmomatic, cutting all reads to a length of 175 bp, resulting in the retention of the maximum number of sequences for all groups. For the COI sequencing run of one group, the ‘repair’ tool was used to retain only contigs with both R1 and R2, and organise them for analysis in Mothur, since the demultiplexing protocol included sequences missing one of the two, causing the pipeline to crash.

### 2.3 DNA Sequence Data Sharing, Bioinformatics and Statistics

Each MZG‐ICE group provided raw (demultiplexed) fastq sequence files for all samples, including replicates, for all gene regions. Sequence data were shared by uploading to an online repository, MZG‐ICE Work‐Area folder, which is accessible only by MZG‐ICE participants. Each group provided data for four gene regions for all 10 samples, with some additional PCR and sequencing replicates. Before uploading and sharing sequence data, the fastq files were examined to confirm correct gene regions and acceptable data quality. The fastq files were uploaded to NCBI SRA BioProject PRJNA1269580.

Samples were processed using Mothur v.1.48.0 (Schloss et al. 2009). The annotated scripts from the pipelines are available at https://github.com/blancobercial/MZG. Sequence reads were assembled and all contigs containing any ambiguity were discarded. All reads shorter than the expected length (depending on the amplicon) were removed. The 18S amplicon sequences were aligned to SILVA 138. COI amplicons were aligned using MAFFT online (Katoh et al. 2017) to a reference dataset downloaded from the MetaZooGene database (https://metazoogene.org/mzgdb/; O'Brien et al. 2024).

Reads were trimmed to the length of the amplicon, and incomplete reads (not starting at base 1 or not reaching the end of the amplicon) were discarded. After this step, the proportion of reads passing these QC steps was analysed, and any sample showing a low retention was scrutinised and pre‐processed to achieve optimal read retention, if possible. After achieving the best retention of sequences based on quality, chimaeras were removed with VSEARCH (Rognes et al. 2016), and single variants were obtained using UNOISE2 (Edgar 2016), as implemented in Mothur using the diffs = 1 setting for denoising.

For 18S rRNA amplicons, rarefaction curves of the number of observed single variants (a proxy for number of taxa, *S*_obs_), Shannon diversity (*H′*), and the Chao1 index were calculated using 1000 randomised iterations to calculate the indices and upper and lower 95% confidence intervals. For COI, single variants were clustered to 95% similarity (a proxy to species level) using the Abundance‐based Greedy Clustering (Edgar 2010; He et al. 2015; Westcott and Schloss 2015) using VSEARCH in Mothur. Then, rarefaction curves on S_obs_, *H′* and Chao1 were calculated following the same approach as for the other amplicons. Finally, taxonomic assignments were done using the naïve classifier as implemented in Mothur, against the MZGdb (O'Brien et al. 2024).

To avoid biases due to unbalanced sequencing depth (Schloss 2024a, 2024b) datasets were rarified for each amplicon to the minimum number of reads that would retain most of the samples from that dataset. All variable reads, including single variants (SV) or OTUs, depending on the amplicon, were retained (i.e., there was no pre‐determined minimal abundance). Diversity indices (*S*_obs_, *H′*, *J'* and Chao1) were then calculated for all samples at the same sequencing depth.

Each dataset was further analysed in PRIMER ver. 7.0.24 with Permanova+ add‐on (Anderson et al. 2008; Clarke and Gorley 2015). Before analysis, samples were standardised by the total and square‐root transformed (i.e., Hellinger transformation), due to the relative abundance nature of the amplicon data (Legendre and Gallagher 2001). A Bray‐Curtis distance similarity matrix was built, and two of the most commonly published graphical representations of distance matrices, Non‐metric Multidimensional Scaling (nMDS) and Principal Coordinates Analysis (PCoA; Gower 1966), were carried out. From the graphical representations, samples representing potential contamination or laboratory artefacts were flagged and analysed in detail at the SV or OTU composition level.

Within‐laboratory replicates were compared across laboratories to measure the role of pure replication versus laboratory effect. To understand the potential effect of biological diversity on the similarity between groups within each location level, a test of the homogeneity of multivariate dispersions (PERMDISP) was carried out to determine whether replicates from locations with lower diversity were more similar to each other than those from samples with higher diversity (Anderson et al. 2006; Gijbels and Omelka 2013).

The number of raw reads passing QC per sample was calculated and graphed for each location and gene. After consideration of sequencing depth, these results were used to identify any locations, laboratories or genes with questionable results. After rarification to a minimum number of reads and Hellinger transformation, Principal Component Analysis (PCoA) and *K*‐means clustering were carried out.

To investigate the potential effect of laboratory choice, individual hierarchical clustering between groups was done for each marker and each location, and a consensus clustering was carried out using quartet topologies in ASTRAL IV (Zhang and Mirarab 2022), as included in ASTER (https://github.com/chaoszhang/ASTER). For hierarchical clustering and dendrogram construction, Bray–Curtis similarity matrices were calculated for each location using data from all groups. Samples with clear signs of contamination or procedural artefacts were excluded at this stage to ensure the dendrograms represented only high‐quality data. The *hclust* function was applied to generate dendrograms for each location, grouping samples by similarity. These dendrograms were converted into phylogenetic trees in Newick format using the *as.phylo* and *write.tree* functions from the *ape* R package (Paradis and Schliep 2018). Location‐specific phylogenetic trees were then combined into a single supertree using ASTER IV software, which synthesised relationships across all input trees to infer the most supported tree while resolving potential conflicts.

Bray–Curtis similarity matrices were calculated for each dataset obtained by the 10 MZG‐ICE research groups across all sampling locations. Pairwise Mantel tests were performed using the Mantel function from the *vegan* R package (Oksanen et al. 2025) to compare the similarity matrices of each group pair, to understand how comparable results were between groups. Ideally, matrices should be highly correlated between the different groups. All matrices were compared, and for each pair, the Pearson correlation method was used to calculate the Mantel statistic, which measures the correlation between the matrices. When matrices for two groups did not have fully matching samples, only the overlapping portion of the matrices was used for the comparison, as implemented using the intersect function. The Mantel test results, including the Mantel statistic and associated *p*‐values, were summarised in a matrix. Samples with signs of contamination or artefacts were excluded from these analyses to ensure robust comparisons.

Heatmaps were prepared for all four genes for the 10 samples analysed by each of the 10 MZG‐ICE research groups. Data are the proportions of the log‐transformed sequence numbers, Log_10_(*x* + 1), for the most abundant taxa. Group #8 had 100% of their reads come back as Arthropoda (Ar), so V4 heatmap used scaling from 0 to 0.52 to prevent the high numbers of Ar from obscuring the other datapoints. Polar charts were plotted for each sample using the same taxonomic groups as the heatmaps. The scaling followed the percentage scale range plotted in the heatmaps.

## 3 Results

The MZG‐ICE results include DNA sequence data for four gene regions sequenced for 10 samples analysed by 10 research groups. The study entailed examination of raw data files, sequence data files after quality control, and resulting sequence numbers, ASVs or OTUs for taxonomic groups identified for the 10 samples. The taxonomic groups selected for analysis included phyla detected by each gene region; COI data were also analysed for identified species of selected taxonomic groups. Sequencing depths between laboratories varied from tens of thousands to several hundreds of thousands of reads per sample. During initial processing and QC, proportions of samples passing QC under a unified pipeline varied widely among groups. When analysed in depth, these differences were resolved by considering: (1) differences in chemistry determining paired‐end (PE) read length during the sequencing (150, 250 or 300 PE); and (2) differences in demultiplexing protocols, some caused by differences in the sequencing protocols. These issues prevented the use of a single, unique pipeline for analysis of all samples and required an additional step for demultiplexing.

**FIGURE 2:**
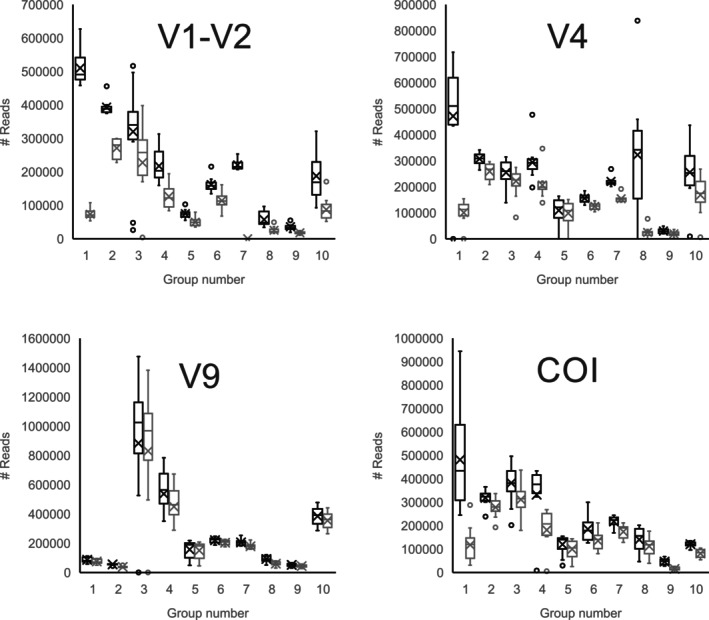
Box‐and‐whisker plot showing the number of reads per marker for each group, before quality control (black) and after quality control (grey). Boxes represent the interquartile range (Q1–Q3), with the median indicated by a horizontal line and the mean marked by an ‘*x*’. Whiskers extend to the maximum value within 1.5 times the interquartile range; outliers beyond this range are shown as individual points (circles).

Due to the diversity of demultiplexing protocols, trimming was done after alignment using the pcr.seqs command in Mothur to trim to the precise region. This approach retained the maximum reads for each group, just as expected if each had been analysed independently. The number of reads per marker for each group differed both before and after quality control (Figure 2). In two cases (i.e., one amplicon for each of two groups), good QC recovery (> 85%) was not achieved due to low quality of the run caused by problems during sequencing, detected as widespread low quality in the ‘per tile sequence quality’ in FastQC (Andrews 2010).

Due to the differences between sequencing depths, a common depth was chosen for each marker to retain the maximum number of samples per marker. Analysed sequencing depths were: 9748 for V1‐V2, 10,000 for V4, 20,000 for V9, and 10,000 for COI. In terms of amplification success, V4 was the marker with the lowest performance: 69 of 100 samples remained after removal of contamination. COI performed slightly better (72 samples), although poor results using standard protocols at some laboratories required use of alternative protocols, including different Taq polymerases, alternative primers, and/or using a PCR‐free library preparation approach (Appendix S1). Success rates for both V1‐V2 and V9 amplification were much higher (88 samples for each marker).

**FIGURE 3:**
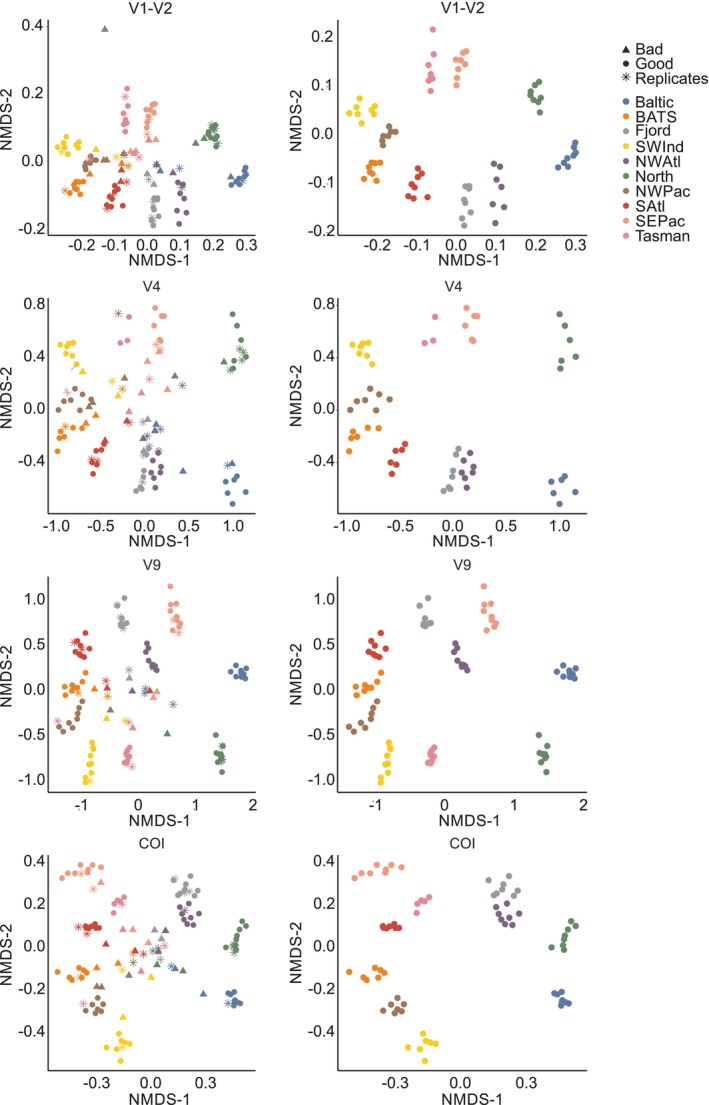
NMDS graphs for the four genetic markers analysed, colour‐coded by collection location, including all samples (left) and after removal of contaminated samples and replicates (right) for each marker.

Based on nMDS graphical analyses from the Hellinger‐transformed, Bray–Curtis similarity matrices, samples from most MZG‐ICE groups clustered by ocean region and biome, including subarctic (e.g., Norwegian fjord and NW Atlantic), subtropical (e.g., BATS, NW Pacific, South Atlantic), and shallow European seas (North and Baltic). However, several samples for each gene marker did not cluster with samples from the same collection location. Samples from one group clustered central to all the others, even separated from the locations, for all markers. One group showed cross‐contamination between samples in three of the markers, and another group had two samples showing cross‐contamination. There were also several instances of mislabeling (Figure 3). Once all outlier data hypothesised to result from mislabeled or contaminated samples were removed, all locations grouped tightly by collection location (Figure 3).

There were significant differences between the dispersion of the samples between locations (V9, *p* < 0.0001; V4, *p* < 0.05; V1‐V2, *p* < 0.005; COI, *p* < 0.0005). In most cases, the Baltic and Tasman Sea samples showed significantly lower dispersion indicating a higher similarity between samples within location. BATS, SW Indian Ocean, and NW Atlantic showed highest dispersion, but many exceptions existed with no significant relationships with diversity for the indices used in this analysis.

**FIGURE 4:**
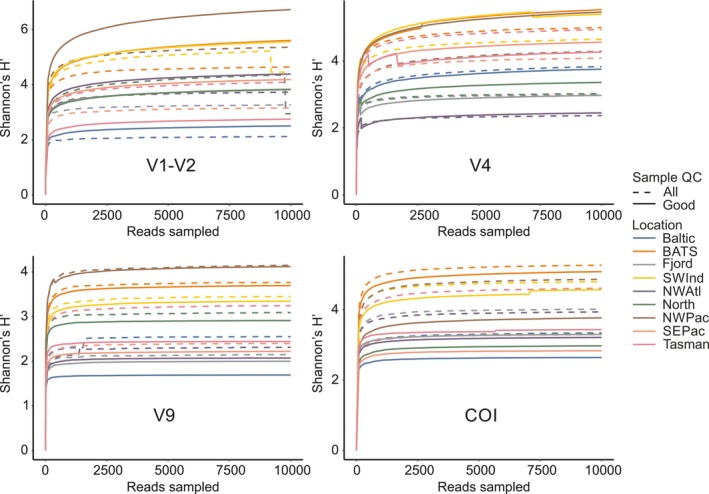
Average Shannon H′ diversity indices per location, obtained before (dashed) and after (continuous line) removal of contaminated samples.

Accumulation curves did not show asymptotes for the species richness or Chao1 index. However, Shannon diversity reached stability for most of the samples and samples with lower total Shannon values reached the asymptotic state more quickly (Figure 4). These results were not unexpected, since there was no minimum number of reads threshold and singletons and doubletons were retained. The results thus agreed with hypothesised expectations of higher diversity indices in samples from subtropical regions and lower diversity in coastal and subarctic samples. There were differences between the 18S rRNA markers and COI; the NW Pacific sample showed much lower COI diversity than other subtropical regions, on par with samples collected outside that region.

**FIGURE 5:**
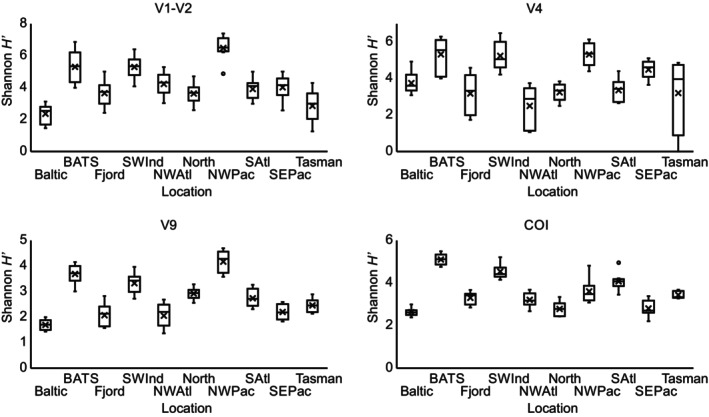
Box and whiskers graphs for Shannon H′ diversity at the minimum shared number of reads for each marker (see text) for each location, using only samples without contamination. Average was represented by an *x*.

**FIGURE 6:**
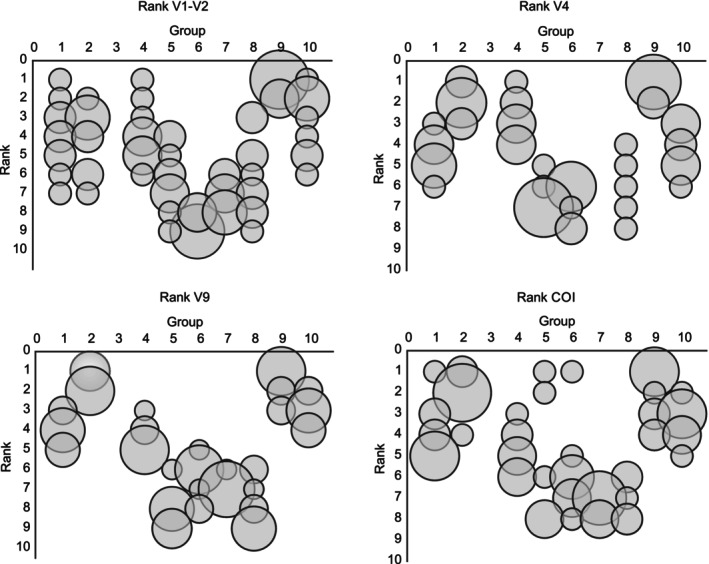
Bubble plot, with bubble size proportional to how many times a Group obtained the highest (1) or lowest (10) diversity for each region, separated by markers. Group #9 was consistently among those that obtained the highest diversity scores for each location and marker.

Results from all MZG‐ICE research groups showed overall similarity in patterns of diversity between samples from different ocean regions, based on the Shannon H′ index (Figure 5). However, there were differences between laboratories in the range of these diversity indices. For example, after standardising to a common number of reads, Group #9 obtained the highest H′ values for 70% of the samples, with the second highest score for another 20% (Figure 6). Results from Group #2 also showed high diversity across multiple samples, while only four of the 40 runs had highest values by any other MZG‐ICE group. After analysing the protocols, Groups #2 and 9 used Q5 High‐Fidelity DNA Polymerase (New England Biolabs, Ipswich, USA) for first round PCR and external sequencing services. No causes for low diversity outcomes could be identified: MZG‐ICE groups that obtained the lowest diversity scores (Groups #5, 6, 7 and 8) did not show any recognisable similarities or commonalities in laboratory protocols or procedures unique to these groups. The effect of contamination and tag jumping on the diversity indices varied depending on the diversity of the sample. In samples with lower diversity, the trend was to increase the measured diversity. However, in samples with high diversity (e.g., NW Pacific or BATS) the effect was to decrease the diversity indices (Figure 4).

**FIGURE 7:**
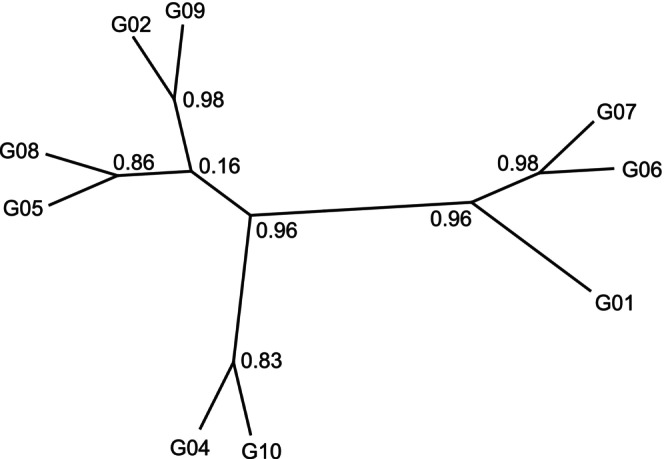
ASTRAL tree obtained after considering the similarity matrices between groups, per location and per marker. Node values indicate the statistical support for that node. Group 3 is not shown.

The reconstructed ASTRAL tree, representing affinities of MZG‐ICE groups based on distance matrices for all four marker genes and all 10 samples, showed a few significantly supported nodes (Figure 7). Groups #2 and 9, which both used Q5 High‐Fidelity DNA Polymerase, formed a strong clade. In contrast, there were no clear similarities in laboratory protocols or methods that may have caused another strong clade with Groups #6 and 7, plus a sister Group #1. Support for the other clades was lower and consistent with expectations for unbiased, parallel analysis of samples in multiple laboratories.

**FIGURE 8:**
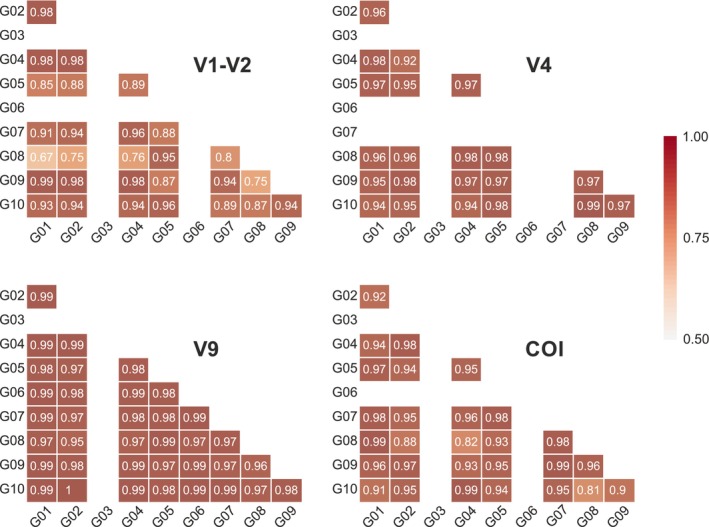
Matrices summarising Mantel tests comparing similarity matrices between laboratories for each marker. All tests indicated positive and significant correlations between labs (white values for each test). In general, highest correlations were obtained for the marker with the lowest diversity (V9), while the lowest correlations were obtained in the marker with the highest diversity (V1, V2).

The patterns of similarities and differences between the samples were overall consistent across all MZG‐ICE groups, with only a few exceptions. Matrices summarising Mantel tests between MZG‐ICE research groups for all gene markers and all samples showed high (> 0.95%) and significant correlations across most samples and groups, with the exception of several MZG‐ICE groups (including #3 and 6), which showed lower correlation scores (Figure 8).

**FIGURE 9:**
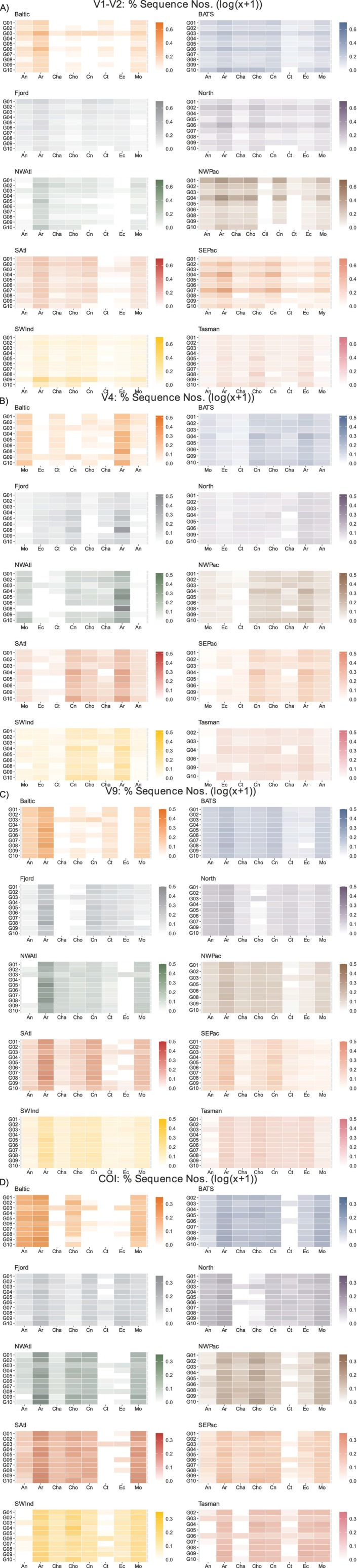
Heatmaps for ocean regions showing proportional sequence numbers (Log 10 (*x* + 1)) for phyla of zooplankton detected by MZG‐ICE groups (G01‐G10). Phylym abbreviations: An = Annelida; Ar = Arthropoda; Cha = Chaetognatha; Cho = Chordata; Cn = Cnidaria; Ct = Ctenophora; Ec = Echinodermata; Mo = Mollusca. (A) 18S rRNA V1‐V2; (B) 18S rRNA V4; (C) 18S rRNA V9; (D) Mitochondrial COI.

Heatmaps prepared for the 10 samples analysed by the 10 MZG‐ICE groups revealed patterns of variation in relative abundances of taxa in eight phyla showing highest sequence numbers (log transformed) for all four gene markers (Figure 9). Overall, V1‐V2 and COI showed lowest values for correlation among MZG‐ICE groups. In contrast, V4 and V9 showed higher correlation among all MZG‐ICE groups (Figure 9).

**FIGURE 10:**
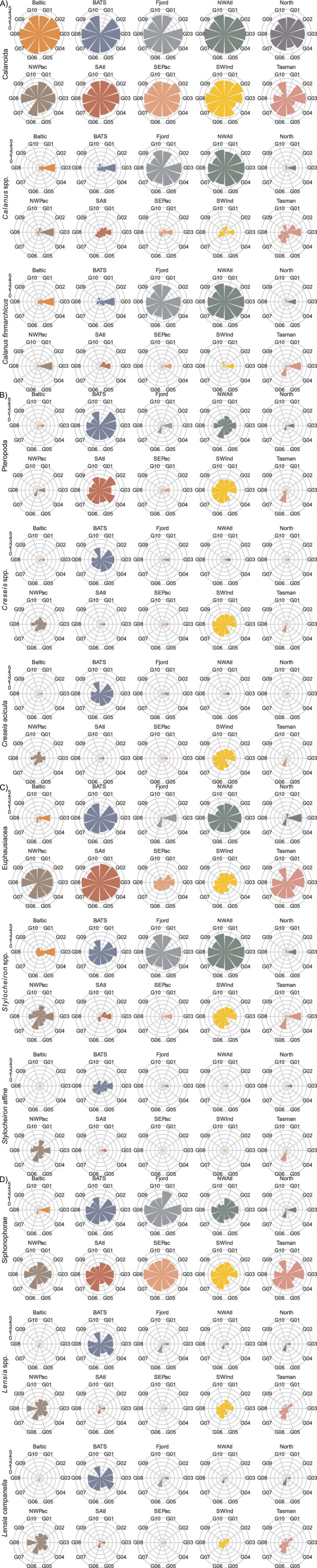
Polar graphs for COI sequence numbers (proportional, log‐transformed) plotted by taxonomic group for (A) Calanoida—*Calanus* spp.— *Calanus finmarchicus* ; (B) Pteropoda—*Creseis* spp.— *Creseis acicula* ; (C) Euphausiacea—*Stylocheiron* spp.— *Stylocheiron affine* ; and (D) Shiphonophorae—Lensia spp.— *Lensia campanella* . The scaling follows the percentage scale range in heatmaps.

Polar graphs confirmed overall patterns of similarity between MZG‐ICE groups for COI metabarcoding results for eight phyla found in highest relative abundances based on sequence numbers (Figure S2). Plots of COI sequence abundances for selected zooplankton taxa showed decreasing similarity among MZG‐ICE groups for different taxonomic levels, from orders to genera to species (Figure 10).

## 4 Discussion

### 4.1 Design and Goals of MZG‐ICE

The MZG‐ICE study was designed to evaluate the use of intercalibration as a primary approach to increasing the use of DNA metabarcoding of zooplankton diversity in the context of ecosystem monitoring, management, assessment and associated policy applications. Molecular protocols were recommended, but the 10 MZG‐ICE research groups were allowed and encouraged to employ protocols, procedures and instrumentation to ensure high‐quality data. The variable protocol chains, with numerous differences between MZG‐ICE laboratories, prevented definitive conclusions about the impacts of each and every protocol step. However, multivariate analysis of MZG‐ICE results allowed detailed examination of the reliability and consistency of patterns of biodiversity resulting from DNA metabarcoding of zooplankton samples carried out in the different laboratories.

The global distribution of MZG‐ICE samples allowed comparison of patterns of variation in different marine biomes, including coastal waters, fjords, regional seas and several ocean basins (Figure 1). The different levels of diversity observed among the samples allowed stronger tests of intercalibration of results from the 10 MZG‐ICE research groups using multiple statistical approaches, including the Shannon diversity index (Figures 4 and 5) and diversity rankings of samples analysed by different MZG‐ICE groups (Figure 6).

DNA metabarcoding provides data for proportional abundances of zooplankton taxa, with differing levels of taxonomic resolution, from phyla to species, depending on the gene marker used (Bucklin et al. 2019, 2022; Ershova et al. 2021; Matthews et al. 2021; Questel et al. 2021). COI and V1‐V2 have been shown to discriminate and identify species, albeit with differences in resolving power between the two markers across the phylogenetic spectrum of the pelagic assemblage (Moutinho et al. 2024). Comparative statistical analysis of taxonomic diversity of the 10 samples, based on proportional sequence numbers for each marker gene and presented using heatmaps and polar plots, allowed identification of the most consistent and reliable results across the 10 MZG‐ICE research groups. COI metabarcoding yielded similar patterns for most of the 10 samples for eight phyla based on heat maps; V9 also showed consistent patterns for these same phyla; more variation among MZG‐ICE groups was evident for both V1‐V2 and V4 (Figure 9).

Detection and identification of zooplankton species were not examined in detail based on MZG‐ICE data. The variation of COI sequence numbers for several species among MZG‐ICE research groups was noteworthy, especially when compared to more consistent and reliable results for the respective genera and orders (Figure 10).

### 4.2 Evaluation of Variation in Results From MZG‐ICE Groups

An overview of intercalibration results was provided by statistical comparison of the similarities and differences of DNA metabarcoding data produced by the 10 MZG‐ICE research groups. The resulting patterns were displayed using various tools, including an ASTRAL tree (Figure 7), which is useful to guide detailed examinations of protocols and procedures that may drive the clustering of MZG‐ICE groups. A Mantel matrix allowed a closer examination of pair‐wise patterns of similarity based on each gene marker (Figure 8). These analyses yielded useful background and perspective for identifying causes of variation between results from different research groups.

Comparison of results from the 10 MZG‐ICE groups, including proportional log‐transformed sequence numbers for different taxonomic levels, from phyla to species, clearly showed the need for multi‐gene DNA metabarcoding for accurate, reliable and relevant analysis of marine zooplankton biodiversity. The V9 and V4 regions of 18S rRNA showed highest consistency in multivariate statistical analyses of biodiversity among MZG‐ICE groups, with higher variability for V1‐V2 and COI. Given the differences in PCR protocols and primers for all gene markers used by MZG‐ICE groups, these findings supported recommendations of the reliability and accuracy of these gene markers for DNA barcoding of zooplankton diversity. These differences may reflect the natural variability of each marker, with V9 most conserved and COI and V1‐V2 most variable, but they may also reflect a higher sensitivity to protocol changes, including reagents, instruments, and individual skills.

One cause of non‐clustering data was hypothesised to be contamination during library preparation, which was indicated by samples showing location‐dominant ASVs/OTUs spread across all other locations, increasing the similarity of samples and affecting both ordination analyses and diversity indices. Contamination appeared to be the cause of the most obvious and significant outlier data for all four gene markers. Results for all 10 MZG‐ICE research groups clustered in nMDS plots by sampling location and ocean region after removal of samples identified as contaminated based on taxonomic composition (Figure 3). Another probable source of variation in results from MZG‐ICE groups was the use of different protocols and reagent kits, including PCR polymerase enzymes and primers, some of which used inosine as a degenerate base, and 1‐step versus 2‐step PCR and library preparation protocols.

An additional finding included the importance of the sequencing platform. Nine of the 10 MZG‐ICE research groups used Illumina MiSeq platforms, including both in their own institutions and external commercial facilities, which yielded data that were comparable after editing for paired‐end (contig) sequence lengths and other parameters. No evident differences could be associated with choice of read lengths (PE‐250, PE‐300 or PE‐350; Appendix S1). One MZG‐ICE group sent samples to an external facility using an Illumina NextSeq sequencing platform, which yielded data with divergent sequence numbers for many taxonomic groups for all marker genes, transfer of endemic ASV/OTUs to all other samples (Figure 3), and unresolvable differences in patterns of variation (Figure 9). These marked differences could have resulted from tag jumping during sequencing using a Next‐Seq platform.

The MZG‐ICE project design included replicate samples selected by each group, including one replicate for their own sample and another of their choice. The replicate samples were sent for sequencing and included in the initial QA/QC evaluations, but not in the definitive analysis. Replicate samples were useful for evaluating outlier data when the replicated sample was of concern, although the evaluation of replicates was not useful for determining causes of outlier data.

The global scope of MZG‐ICE provided further evidence of the need for taxonomically and geographically complete multi‐gene reference sequence databases for marine ecosystems, including accurate species identification of voucher specimens by morphological taxonomic experts and inclusion of complete collection metadata (Bucklin et al. 2021; Rimet et al. 2021; Peters et al. 2025; Questel et al. 2025). The MetaZooGene Atlas and Database (Bucklin et al. 2021; O'Brien et al. 2024; https://metazoogene.org/database) used for classification and identification of sequences for this study is particularly useful due to the inclusion of collection georeferencing (latitude and longitude coordinates) and specification of ocean regions for barcoded specimens. The linked database and atlas functions of MZGdb allow search and analysis of biodiversity, including assessment of completeness, for both taxonomic groups and ocean regions, which improves the accuracy of identification of species, especially in regions with limited numbers of barcoded species (Bucklin et al. 2021; O'Brien et al. 2024).

### 4.3 Recommendations for DNA Metabarcoding of Zooplankton Diversity

MZG‐ICE results indicated essential elements of the metabarcoding analytical chain:

- Detailed recording of all protocols, reagents and methods used from DNA to data.
- Immediate submission of sequence data to public open‐access online databases.
- Screening each dataset separately when analysing samples from different sources/groups, in order to understand the structure of the raw files (e.g., presence/absence of primers, tails).
- Pre‐treating each dataset from different sources/groups to ensure unbiased QC.
- Centralised data QA/QC using same parameters and benchmarks for data finalisation.
- Inclusion of replicates for all analytical steps.
- Multidimensional analysis to detect outliers, including contamination and errors.
- Creation and distribution of standard mock samples to participating laboratories.

## 5 Conclusions

The MetaZooGene Intercalibration Experiment (MZG‐ICE) examined patterns of variation in resulting data and biodiversity estimates based on DNA metabarcoding of zooplankton samples carried out by 10 different laboratories and research groups. The option for each of the 10 MZG‐ICE groups to use reagents, protocols and procedures of their choosing was designed to increase the likelihood of success for all groups at every analytical step from DNA to data. The resulting DNA sequence data were uploaded to a shared repository for centralised QA/QC, bioinformatics and statistics analysis. Methodological variation among MZG‐ICE laboratories was consistent with and essential to the design of intercalibration experiments, which by definition do not require or expect standardisation or selection of best practices. MZG‐ICE provided evidence of the reliability and reproducibility of DNA metabarcoding results from diverse research groups for zooplankton samples collected in different ocean regions and biomes. MZG‐ICE conclusions supported the overarching goal of encouraging and facilitating the integration of DNA metabarcoding of zooplankton biodiversity into routine monitoring and management of marine ecosystems.

## Author Contributions

All authors contributed to the research described in this manuscript. Contributions include: designed research (A.B., R.E., T.F., J.H., J.A.H., P.M.A., L.S., A.W.‐Z., L.B.‐B. and P.G.B.‐L.), performed research (P.G.B.‐L., E.D., E.E.‐M, C.E.G., A.G., J.G., S.K., M.M., A.M.P., R.R.‐P., S.D. and K.T.C.A.P.), analysed data (L.B.‐B., R.R.‐P. and J.M.Q.) and wrote the paper (A.B., L.B.‐B. and J.M.Q.).

## Funding

Funding was provided by the Scientific Committee on Oceanic Research (SCOR) and the US National Science Foundation (NSF Grant OCE‐1840868) and by National SCOR Committees. Additional support and funding were provided by The BIOS‐SCOPE program of the Simons Foundation International provided funding to LBB. Australian Antarctic Division provided funding for sample processing (Australian Antarctic Science Project, AAS 4556). Environmental Research and Technology Development Fund (JPMEERF20224R03) provided funding to JH. Millennium Institute of Oceanography (ANID, AIM23‐0003) provided support for sequencing the Southeastern Pacific samples. Naturalis Biodiversity Center provided funding for sample processing and DNA sequencing. This is publication number 110 from the Senckenberg am Meer Molecular and Metabarcoding Laboratory. University of Gdańsk (Poland) provided support for collection of the Baltic Sea sample during a field expedition of the R/V *Oceanograf*. University of Connecticut, Center for Genomic Innovation (CGI), provided expert technical assistance and analytical support for molecular analysis of MZG‐ICE samples. The Institute of Marine Research, Norway, provided support for collection of samples from the North Sea and Norwegian fjords. The Norwegian Research Council (CoastRisk 299554/F40) provided funding for EE.

## Disclosure

Benefit‐Sharing Statement: A research collaboration was developed with scientists from countries providing genetic samples; all collaborators are included as co‐authors. The results of research have been shared with the provider communities and the broader scientific community. The research addresses a priority concern, including the accurate identification and conservation of marine organisms in the global ocean. More broadly, our group is committed to international scientific partnerships, as well as institutional capacity building.

## Conflicts of Interest

The authors declare no conflicts of interest.

## Supporting information


**Appendix S1:** men70090‐sup‐0001‐AppendixS1.docx.

**Appendix S2:** men70090‐sup‐0002‐AppendixS2.docx.

## Data Availability

Data Accessibility Statement: Genetic data have been deposited in the NCBI SRA (BioProject PRJNA1269580). All data will be released for public access upon acceptance for publication. Scripts (both Mothur and R) are available at https://github.com/blancobercial/MZG.
