# Supplementary material for: MetaZooGene Intercalibration Experiment (MZG‐ICE): Metabarcoding Marine Zooplankton Diversity of the Global Ocean
Source: Mol Ecol Resour. 2025 Dec 26;26(1):e70090. doi: 10.1111/1755-0998.70090 (PMC12742283; doi:10.1111/1755-0998.70090)
Supplement: Supplementary file 1 — Appendix S1: men70090‐sup‐0001‐AppendixS1.docx. [file MEN-26-e70090-s002.docx]

**MetaZooGene Intercalibration Experiment (MZG-ICE):**

**Metabarcoding Marine Zooplankton Diversity of the Global Ocean**

Leocadio Blanco-Bercial et al.

Submitted to Molecular Ecology Resources (November 24, 2025)

**Suppl. File 1. Work flow and protocol details for analysis of zooplankton samples by MZG-ICE research groups, identified by group number and home institution.**

**Group 1. University of Connecticut (USA)**

DNA extraction reference: Bucklin et al. (2022)

DNA amount: 1 µg (dry)

Workflow: For all gene regions, PCR primers were altered for multiplexed sequencing by adding 5’ adapters (Illumina, Inc., San Diego, CA) for first PCR: Forward: 5’-TCGTCGGCAGCGTCAGATGTGTATAAGAGACAG-3’ and Reverse: 5’-GTCTCGTGGGCTCGGAGATGTGTATAAGAGCAG-3’

PCR Amplifications for all 4 gene regions (except as noted):

Taq and buffer used: KAPA HiFi for V1-V2, V4 and V9; Platinum Taq for COI

Template DNA per sample: 20ng

PCR: 2 steps

Illumina chemistry: V3

Illumina PE for V1-V2, V4, and V9: 2x250; COI: 2X300

V1-V2 PCR used forward primer SSU_F04 and reverse primer SSU_R22 (Fonseca et al., 2010. PCR protocol: one denaturation cycle at 95°C for 2 min; 35 cycles of 95°C for 1 min, 57°C for 45 sec, 72°C for 3 min; a single extension cycle of 72°C for 10 min; and an infinite hold at 4°C.

V4 PCR used forward primer TAR euk454FWD1 and reverse primer TAR eukREV3 (Stoeck et al., 2010). PCR protocol: one denaturation cycle at 95°C for 2 min; 10 cycles of 95°C for 20 sec, 57°C for 30 sec, 72°C for 15 sec; 15 cycles of 98°C for 10 sec, 66°C for 30 sec, 72°C for 15 sec; extension cycle of 72°C for 7 min; hold at 4°C.

V9 PCR used forward primer 1389F and reverse primer 1510R (Amaral-Zettler et al., 2009). PCR protocol: one denaturation cycle at 95°C for 30 sec; 10 cycles of 98°C for 20 sec, 56°C for 30 sec, 72°C for 10 sec; 15 cycles of 98°C for 10 sec, 66°C for 30 sec, 72°C for 15 sec; extension cycle of 72°C for 7 min; hold at 4°C.

COI PCR used forward primer mtCOIintF (Leray et al., 2013) and reverse primer jghCO2198 (Geller et al., 2013). PCR protocol: one denaturation cycle at 94°C for 1 min; 35 cycles of 94°C for 10 min, 46°C for 30 sec, 72°C for 1 min; extension cycle of 72°C for 5 min; hold at 4°C.

2nd PCR protocol: Nextera (external)

Own laboratory / External: Library prep. in laboratory; external sequencing

Platform: MiSeq

**Group 2. Bermuda Institute of Ocean Sciences (Bermuda)**

DNA extraction reference: Blanco-Bercial (2020)

DNA amount: 1 µg (dry)

Own laboratory / External: Library preparation and sequencing done at external facility (Rochester University URGenomics).

PCR amplification for all gene regions:

Taq and buffer: Q5 High-Fidelity DNA Polymerase

Template ngDNA per sample: 5ng

V1-V2 (Fonseca et al., 2010):

30 s at 98 °C, 35 cycles of 98°C for 30s, 57 °C for 30 s, and 72°C for 30 s, 10 min at 72°C.

V4 (Stoeck et al., 2010):

30 s at 98 °C, followed by 10 cycles of 30 s at 98◦C, 30 s at 53◦C and 30 s at 72◦C, and then 15 cycles of 10 s at 98◦C, 30 s at 48◦C and 30 s at 72◦C, and a final elongation step at 72◦C for 10 min

V9 (Amaral-Zettler et al., 2009):

98 C for 30 s, 35 cycles of 98°C for 30 s, 63°C for 30 s, and 30 s at 72◦C, and a final elongation step at 72◦C for 10 min.

COI (Leray et al., 2013):

30 s at 98°C, 35 cycles of 98°C for 30s, 62°C for 30 s, and 72°C for 30 s, 10 min at 72°C.

1 step or 2 step PCR: 2 step

2nd PCR protocol: Nextera

Platform: MiSeq

Illumina chemistry: V3

Illumina PE300 for V1V2, V4, COI. PE150 for V9.

**Group 3. University of Concepción (Chile)**

DNA extraction reference: Blanco-Bercial (2020)

DNA amount: 60 ng (dry)

Workflow: Ten lyofilized DNA samples were reconstituted in 20μL of 10mM Tris, brought to the same concentration before PCR amplification .. DNA concentrations were quantified using fluorometry (Qubit dsDNA HS Assay Kit; Qubit 4.0) before library preparation. The 260/280 ratio was measured (NanoQuant InfiniteM200Pro from TECAN) to verify sample purity using 2μL of the sample.

PCR Amplifications for all 4 gene regions (except as noted):

Taq and buffer used: Platinum SuperFi II Polymerase HotStart ReadyMix (Thermo Fisher)

Template ngDNA per sample: 1 ng for reactions of 12.5 ul with primer

PCR: 2 steps

Illumina chemistry: V3

Illumina PE: 300

V1-V2: Primers SSU_FO4 (5′-GCTTGTCTCAAAGATTAAGCC-3′) and SSU_R22 (5′- GCCTGCTGCCTTCCTTGGA-3′) were used to amplify approximately 450 bp of the V1–V2 regions (Fonseca et al. 2010). The first PCR protocol followed these steps: one denaturation cycle at 98°C for 30 sec; 38 cycles of 98°C for 10 sec, 60°C for 10 sec, 72°C for 30 sec; a single extension cycle of 72°C for 5 min; and an infinite hold at 4°C.

V4: Primers TAReuk454FWD1 (5′-CCAGCASCYGCGGTAATTCC-3′) and TAReukREV3 (5′- ACTTTCGTTCTTGATYRA-3′) were used to amplify the V4 region (Stoeck et al., 2010). The first PCR protocol was: one denaturation cycle at 98°C for 30 sec; 38 cycles of 98°C for 10 sec, 60°C for 10 sec, 72°C for 30 sec; a single extension cycle of 72°C for 5 min; and an infinite hold at 4°C.

V9: Primers 1389 V9 forward (5′-TTGTACACACCGCCC-3′) and 1510 V9 reverse (5′- CCTTCYGCAGGTTCACCTAC-3′) were used to amplify the V9 region (Amaral-Zettler et al., 2009). The first PCR protocol: one denaturation cycle at 98°C for 30 sec; 38 cycles of 98°C for 10 sec, 60°C for 10 sec, 72°C for 30 sec; a single extension cycle of 72°C for 5 min; and an infinite hold at 4°C.

COI: Primers mlCOIintF (5′-GGWACWGGWTGAACWGTWTAYCCYCC-3′) and jgHCO2198 (5′- TAIACYTCIGGRTGICCRAARAAYCA-3′) were used to amplify COI (Leray et al., 2013). The first PCR protocol: one denaturation cycle at 98°C for 30 sec; 36 cycles of 98°C for 10 sec, 55°C for 10 sec, 72°C for 30 sec; a single extension cycle of 72°C for 5 min; and an infinite hold at 4°C.

2nd PCR protocol: Oligos as in Fadrosh et al. (2014)

Own laboratory / External: Library preparation and sequencing done at external facility

Platform: NextSeq

Pools were re-amplified with a second PCR using the Platinum SuperFi II Polymerase HotStart ReadyMix (Thermo Fisher) to add the Illumina P5 and P7 adapters, using the Illumina Nextera XT Index Kit v2. The amplification protocol was the same for all four molecular markers: one denaturation cycle at 98°C for 2 min; 6 cycles of 98°C for 30 sec, 55℃ for 30 sec and 72°C for 30 sec; final extension cycle of 72°C for 5 min; hold at 4°C.

The libraries were then purified with Agencourt AMPure XP magnetic beads (Beckman Coulter) at 0.8X ratio and eluted in 10 mM Tris. Library size was verified by capillary electrophoresis using the Fragment Analyzer system with the DNF-910 Kit (Agilent).

Libraries were diluted to 4 nM and pooled. Sequencing used the recommended manufacturer's protocol (MiSeq System Denature and Dilute Libraries Guide). Libraries were denatured, diluted (MiSeq Reagent Kit v3 600 cycles), and mixed with PhiX control libraries at 20% to introduce heterogeneity to the sequencing. Sequencing was carried out at the AUSTRAL-omics laboratory, which provided FastQ configuration and paired-end readings, using the NextSeq platform.

# Group 4. German Centre for Marine Biodiversity Research (Germany)

DNA extraction reference: Rossel et al. (2023)

DNA amount: 300 ng (dry)

Workflow: The first PCR used recommended primers for each gene region with Illumina adaptors: Forward: ACACTCTTTCCCTACACGACGCTCTTCCGATCT + Locus primer; Reverse: GTGACTGGAGTTCAGACGTGTGCTCTTCCGATCT + Locus primers.

For the second PCR, unique Nextera double index (i5, i7) adapters were added: Forward: 5’-CAAGCAGAAGACGGCATACGAGAT[i7]GTCTCGTGGGCTCGG-3’; Reverse: 5’-AATGATACGGCGACCACCGAGATCTACAC[i5]TCGTCGGCAGCGTC-3’. PCR products were pooled separately for each fragment based on Relative Florescent Unit values for normalization. Subsequent steps included: purification (AmpureXP); library quantification (Collibri quantification kit).

PCR Amplifications for all 4 gene regions (except as noted):

Taq and buffer used: SsoAdvanced Universal IT SYBR Green Supermix

Template ngDNA per sample: 10-15ng

PCR: 2 steps

Illumina chemistry: V3

Illumina PE: 300

V1-V2 PCRs used forward primer SSU_F04 and reverse primer SSU_R22 (Fonseca et al., 2010. The first PCR protocol was: one denaturation cycle at 98°C for 3 min; 30 cycles of 98°C for 30 sec, 57°C for 30 sec, 72°C for 30 sec; a single extension cycle of 72°C for 3 min; a melt curve with temperature rising from 50°C to 95°C in 0.5°C increments every 5 sec; and an infinite hold at 5°C.

V4 PCRs used forward primer TAR euk454FWD1 and reverse primer TAR eukREV3 (Stoeck et al., 2010). The first PCR protocol was: one denaturation cycle at 98°C for 3 min; 35 cycles of 98°C for 30 sec, 54°C for 30 sec, 72°C for 30 sec; a melt curve with temperature rising from 72°C to 95°C in 0.5°C increments every 5 sec; hold at 5°C.

V9 PCRs used forward primer 1389F and reverse primer 1510R (Amaral-Zettler et al., 2009). The first PCR protocol was: one denaturation cycle at 98°C for 3 min; 30 cycles of 98°C for 30 sec, 57°C for 30 sec, 72°C for 30 sec; a melt curve with temperature rising from 50°C to 95°C in 0.5°C increments every 5 sec; hold at 5°C.

COI PCRs used forward primer mtCOIintF (Leray et al., 2013) and reverse primer jghCO2198 (Geller et al., 2013). The first PCR protocol was: one denaturation cycle at 98°C for 3 min; 35 cycles of 98°C for 3 min, 54°C for 30 sec, 72°C for 30 sec; extension cycle of 72°C for 3 min; a melt curve with temperature rising from 72°C to 95°C in 0.5°C increments every 5 sec; hold at 5°C.

The second PCR used the same protocol for all four gene regions: one denaturation cycle at 98°C for 3 min; 10 cycles of 98°C for 30 sec, 72°C for 30 sec; extension cycle of 72°C for 5 min; a melt curve with temperature rising from 50°C to 95°C in 0.5°C increments every 5 sec; hold at 5°C.

2nd PCR protocol: Nextera

Own laboratory / External: All procedures in the PI laboratory

Platform: MiSeq

**Group 5. Australian Antarctic Division (Australia)**

DNA extraction reference: Suter et al. (2021)

DNA amount:130 ng (dry)

Workflow: Amplicon libraries for Illumina MiSeq sequencing were produced using 2-stage PCR. First PCR used Illumina adapter sequences: Forward: 5’-TCGTCGGCAGCGTCAGATGTGTATAAGAGACAG-3’; Reverse: 5’-GTCTCGTGGGCTCGGAGATGTGTATAAGAGACAG-3’, with 6-7 bp MIDs (Meyer et al., 2008) and locus-specific primer sequences added at 3’ end. Second PCRs used Illumina P5/P7 flow cell adaptors and 8 base pair indices (Faircloth & Glenn, 2012): P5: 5'-AATGATACGGCGACCACCGAGATCTACAC [i5index] TCGTCGGCAGCGTC-3’; P7:5’-CAAGCAGAAGACGGCATACGAGAT [i7index] GTCTCGTGGGCTCGG-3’

Taq polymerase used: Ampli Taq 360 Master Mix

Template ngDNA per sample: 5 ng

1 step or 2 step PCR: 2 step PCR

Illumina chemistry: V3

Illumina: V1-V2: PE 300; V4, V9, COI: PE 280

V1-V2 PCRs used forward primer SSU_F04 and reverse primer SSU_R22 (Fonseca et al., 2010. The first PCR protocol was: one denaturation cycle at 95°C for 10 min; 35 cycles of 95°C for 30 sec, 53°C for 30 sec, 72°C for 30 sec; a single extension cycle of 72°C for 7 min.

V4 PCRs used forward primer TAR euk454FWD1 and reverse primer TAR eukREV3 (Stoeck et al., 2010). The first PCR protocol was: one denaturation cycle at 95°C for 10 min; 10 cycles of 95°C for 30 sec, 44°C for 30 sec, 72°C for 30 sec; 20 cycles of 95°C for 30 sec, 62°C for 30 sec, 72°C for 30 sec; extension cycle of 72°C for 7 min.

V9 PCRs used forward primer 1389F and reverse primer 1510R (Amaral-Zettler et al., 2009). PCR protocol was: one denaturation cycle at 95°C for 10 min; 35 cycles of 95°C for 30 sec, 55°C for 30 sec, 72°C for 30 sec; extension cycle of 72°C for 7 min.

COI PCRs used forward primer mtCOIintF (Leray et al., 2013) and reverse primer jghCO2198 (Geller et al., 2013). PCR protocol was: one denaturation cycle at 95°C for 10 min; 16 cycles of 95°C for 30 sec, 62°C for 30 sec (subtracting 1°C each cycle), 72°C for 30 sec; 25 cycles of 95°C for 30 sec, 55°C for 30 sec, 72°C for 30 sec; extension cycle of 72°C for 7 min.

The second PCR used the same protocol for all four gene regions: one denaturation cycle at 95°C for 30 sec; 10 cycles of 95°C for 30 sec, 55°C for 30 sec; 72°C for 30 sec; extension cycle of 72°C for 7 min; hold at 4°C.

The size of the final library was verified using a Bioanalyzer DNA 1000 chip. DNA amplicon sizes were determined using an Agilent Technologies 2100 Bioanalyzer. The raw sequences from the paired-end sequencing were identified based on the i5/i7 tag. The de-multiplexed reads were further filtered based on identification of correct first round MID tag, which were subsequently trimmed off. Sequences without correct first round MID tags were discarded.

2nd PCR protocol: Oligos as in Faircloth and Glenn (2012)

Own laboratory / External: Library preparation in PI laboratory, external sequencing

Platform: MiSeq

**Group 6. University of Tokyo (Japan)**

DNA extraction reference: Hirai et al. (2015)

DNA amount: 10 µg (dry)

Workflow: The first PCR consisted of three technical replicates, with all PCR products pooled before purification using Ampure XP. The purified pooled products were then used as templates for the second PCR. After amplification, the second PCR products were pooled, purified with Ampure XP, and quantified via qPCR before sequencing on the Illumina MiSeq.

1st PCR primers with adaptor sequences were: Forward: ACACTCTTTCCCTACACGACGCTCTTCCGATCT + region specific primer; Reverse: GTGACTGGAGTTCAGACGTGTGCTCTTCCGATCT + region specific primer.

Taq and buffer: KOD One (TOYOBO)

Template DNA per sample: 5ng

1 step or 2 step PCR: 2 steps

Illumina chemistry: V3

Illumina: PE 300

V1-V2 PCR used forward primer SSU_F04 and reverse primer SSU_R22 (Fonseca et al., 2010). The PCR protocol was: one denaturation cycle at 94°C for 1 min; 30 cycles of 98°C for 10 sec, 57°C for 5 sec, 68°C for 10 sec; a single extension cycle of 68°C for 2 min; and an infinite hold at 4°C.

V4 PCR used forward primer TAR euk454FWD1 and reverse primer TAR eukREV3 (Stoeck et al., 2010). PCR protocol was: one denaturation cycle at 94°C for 1 min; 30 cycles of 98°C for 10 sec, 55°C for 5 sec, 68°C for 10 sec; extension cycle of 68°C for 2 min; hold at 4°C.

V9 PCR used forward primer 1389F and reverse primer 1510R (Amaral-Zettler et al., 2009). PCR protocol was: one denaturation cycle at 94°C for 1 min; 30 cycles of 98°C for 10 sec, 56°C for 5 sec, 68°C for 10 sec; extension cycle of 68°C for 2 min; hold at 4°C.

COI PCR used forward primer mtCOIintF (Leray et al., 2013) and reverse primer jghCO2198 (Geller et al., 2013). PCR protocol was: one denaturation cycle at 94°C for 1 min; 35 cycles of 98°C for 10 sec, 46°C for 5 sec, 68°C for 10 sec; extension cycle of 68°C for 2 min; hold at 4°C.

2nd PCR protocol:

Primers for the second PCR were:

Forward: AATGATACGGCGACCACCGAGATCTACAC[i5]ACACTCTTTCCCTACACGACGC;

Reverse: CAAGCAGAAGACGGCATACGAGAT[i7]GTGACTGGAGTTCAGACGTGTG

Own laboratory / External: All procedures in the PI laboratory

Platform: MiSeq

**Group 7. University of Gdańsk (Poland)**

DNA extraction reference: TBD

DNA amount: 1 µg (in 10 µL water)

Workflow: For all gene regions, PCR primers were altered for multiplexed sequencing by adding 5’ adapters (Illumina, Inc., San Diego, CA): Forward: 5′-TCGTCGGCAGCGTCAGATGTGTATAAGAGACAG-3′; Reverse: 5′-GTCTCGTGGGCTCGGAGATGTGTATAAGAGACAG-3′

Template ngDNA per sample: 20 ng; North and Baltic samples used 10 ng

1 step or 2 step PCR: 2 step PCR

Illumina chemistry: V3

Illumina PE 300

V1-V2 PCR used forward primer SSU_F04 and reverse primer SSU_R22 (Fonseca et al., 2010. The first PCR protocol was: one denaturation cycle at 95°C for 5 min; 35 cycles of 95°C for 30 sec, 49°C for 30 sec, 72°C for 90 sec; a single extension cycle of 72°C for 7 min.

V4 PCR used forward primer TAR euk454FWD1 and reverse primer TAR eukREV3 (Stoeck et al., 2010). The first PCR protocol was: one denaturation cycle at 95°C for 3 min; 35 cycles of 95°C for 30 sec, 52°C for 30 sec, 72°C for 30 sec; extension cycle of 72°C for 5 min.

V9 PCR used forward primer 1389F and reverse primer 1510R (Amaral-Zettler et al., 2009). The first PCR protocol was: one denaturation cycle at 95°C for 3 min; 35 cycles of 95°C for 30 sec, 59°C for 30 sec, 72°C for 30 sec; extension cycle of 72°C for 5 min.

COI PCR used forward primer mtCOIintF (Leray et al., 2013) and reverse primer jghCO2198 (Geller et al., 2013). The first PCR protocol was: one denaturation cycle at 95°C for 3 min; 35 cycles of 95°C for 30 sec, 45°C for 30 sec, 72°C for 90 sec; extension cycle of 72°C for 5 min.

2nd PCR protocol: Nextera

Own laboratory / External: External facility: commercial sequencing laboratory (Genomed, Poland).

Platform: MiSeq

**Group 8. Institute of Marine Research (Norway)**

DNA extraction reference: Ershova et al. (2023)

DNA amount: 1.39 µg (in buffer)

Workflow: Taq and buffer: AmpliTaq Gold for PCR1; HotStarTaq for PCR2 (Qiagen Multiplex PCR kit)

Template ngDNA per sample: 10-30 ng

1 step or 2 step PCR: 2 step for 18S, 1 step for COI

Illumina chemistry: V3 for COI, 18S V1/V2 and 18S V4; V2 for 18S V9

Illumina PE 300; PE 250 for 18S rRNA V9

V1-V2 PCR used forward primer SSU_F04 and reverse primer SSU_R22 (Fonseca et al., 2010). First PCR protocol: one denaturation cycle at 95°C for 5 min; 10 cycles of 95°C for 10 sec, 65°C for 30 sec. 72°C for 30 sec; a single extension cycle of 72°C for 5 min.

V4 PCR used forward primer TAR euk454FWD1 and reverse primer TAR eukREV3 (Stoeck et al., 2010). First PCR protocol: one denaturation cycle at 95°C for 5 min; 10 cycles of 95°C for 10 sec, 65°C for 30 sec. 72°C for 30 sec; a single extension cycle of 72°C for 5 min.

V9 PCR used forward primer 1389F and reverse primer 1510R (Amaral-Zettler et al., 2009).

First PCR protocol: one denaturation cycle at 95°C for 5 min; 10 cycles of 95°C for 10 sec, 65°C for 30 sec. 72°C for 30 sec; a single extension cycle of 72°C for 5 min.

COI PCR used forward primer mtCOIintF (Leray et al., 2013) and reverse primer mlCOIintF-XT (Wangensteen et al., 2018) with individual 8 b.p. sample tags (same tag on forward and reverse primers). PCR protocol: 10 min at 95 °C, followed by 35 cycles of 94 °C for 1 min, 45 °C for 1 min, and 72 °C for 1 min, and a final extension of 5 min at 72 °C PCR products pooled and cleaned via Qiagen Minelute DNA Purification Kit, quantified using Qubit dsDNA HS Assay Kit prior to library preparation.

Library preparation for V1-V2, V4, and V9 used Illumina adapters and i5/i7 indexes. Quantification was done using Qubit dsDNA HS Assay Kit and size selection using Blue Pippin. V1-V2 and V4 were sequenced on the same V3 2x300 kit (each library was allocated 1/8 of kit). V9 was sequenced on V2 2x250 Nano Kit.

Library preparation for COI ligation based (NEXT-Flex PCR free kit), size-selection using AMPure XP beads (Beckman Coulter), and the final concentration of the libraries was measured via qPCR with the SparQ Universal Library Quantification Kit (Quantabio). Sequenced on 1/8 of a V3 2x300 kit.

2nd PCR protocol: Nextera (18S); NEXT-Flex PCR free (COI)

Own laboratory / External: All procedures in the PI laboratory

Platform: MiSeq

**Group 9. Oceanographic Research Institute (South Africa)**

DNA extraction reference: Singh et al. (2021)

DNA amount: 1.2 µg (dry)

Workflow: The PCR machine was pre-heated for Q5® High-Fidelity DNA Polymerase (New England Biolabs). The first PCRs were carried out in triplicate and then pooled. The second PCR, purification, and library preparation were done at the preferred sequencing facility

Taq and buffer: Q5 High-Fidelity DNA Polymerase

Template ngDNA per sample: 5ng

1 step or 2 step PCR: 1 step

Illumina chemistry: V2

Illumina PE 250

V1-V2 PCR used forward primer SSU_F04 and reverse primer SSU_R22 (Fonseca et al., 2010. The PCR protocol was: one denaturation cycle at 98°C for 10 min; 25 cycles of 98°C for 1 min, 57°C for 1 min, 72°C for 1 min; a single extension cycle of 72°C for 4 min.

V4 PCR used forward primer TAR euk454FWD1 and reverse primer TAR eukREV3 (Stoeck et al., 2010). PCR protocol was: one denaturation cycle at 98°C for 10 min; 25 cycles of 98°C for 1 min, 57°C for 1 min, 72°C for 1 min; extension cycle of 72°C for 4 min.

V9 PCR used forward primer 1389F and reverse primer 1510R (Amaral-Zettler et al., 2009). PCR protocol was: one denaturation cycle at 98°C for 10 min; 25 cycles of 98°C for 1 min, 57°C for 1 min, 72°C for 1 min; extension cycle of 72°C for 4 min.

COI PCR used forward primer mtCOIintF (Leray et al., 2013) and reverse primer HCO2198 (Folmer et al., 1994). PCR protocol was: one denaturation cycle at 98°C for 30 sec; 25 cycles of 98°C for 10 sec, 46°C for 30 sec, 72°C for 30 sec; extension cycle of 72°C for 4 min.

2nd PCR protocol: Nextera (external)

Own laboratory / External: external facility

Platform: MiSeq

**Group 10. Naturalis Biodiversity Center (The Netherlands)**

DNA extraction reference: TBD

DNA amount: 3.1 µg (in water)

Workflow: After the first PCR, a 0.9X bead clean-up was performed for the V1-V2, V4, and COI regions, using NucleoMag NGS magnetic Clean-up and size selection beads; a 1X bead clean-up was used for V9. The first and second PCRs used the same protocol, except that for the second PCR, unique IDT10 label combinations were added to the primers and 8 PCR cycles were used.

Taq and buffer: Taqman Environmental MasterMix; for COI: Amplitaq Gold PCR MasterMix

Template ngDNA per sample: 1 ul per sample; for COI: 2.5 ul per sample:

1 step or 2 step PCR 2 step PCR

Illumina chemistry: V3, 2x 300 bp

MiSeq Illumina sequencing: PE #?

V1-V2 PCR used forward primer SSU_F04 and reverse primer SSU_R22 (Fonseca et al., 2010. The PCR protocol was: one denaturation cycle at 95°C for 10 min; 35 cycles of 95°C for 15 sec, 50°C for 30 sec, 72°C for 40 sec; a single extension cycle of 72°C for 5 min; and an infinite hold at 12°C.

V4 PCR used forward primer TAR euk454FWD1 and reverse primer TAR eukREV3 (Stoeck et al., 2010). The PCR protocol was: one denaturation cycle at 95°C for 10 min; 35 cycles of 95°C for 15 sec, 50°C for 30 sec, 72°C for 40 sec; a single extension cycle of 72°C for 5 min; and an infinite hold at 12°C.

V9 PCR used forward primer 1389F and reverse primer 1510R (Amaral-Zettler et al., 2009). The PCR protocol was: one denaturation cycle at 95°C for 10 min; 35 cycles of 95°C for 15 sec, 50°C for 30 sec, 72°C for 40 sec; a single extension cycle of 72°C for 5 min; and an infinite hold at 12°C.

COI PCR used forward primer mtCOIintF (Leray et al., 2013) and reverse primer jghCO2198 (Geller et al., 2013). PCR protocol was: one denaturation cycle at 95°C for 10 min; 35 cycles of 94°C for 1 min, 45°C for 1 min, 72°C for 1 min; extension cycle of 72°C for 5 min; hold at 12°C.

Library preparation: To determine concentrations, all 14 samples for each 18S region (V1-V2, V4, V9) were measured on a Fragment Analyzer (FA) using the dsDNA 810 kit and were then equimolarly pooled. The COI sample concentrations were measured on the TapeStation (D5000 Screentape) before equimolar pooling. A bead clean-up was performed on all four pools using NucleoMag NGS magnetic Clean-up and size selection beads.

2nd PCR protocol: IDT 10 for Illumina

Own laboratory / External: All procedures at an external facility

Platform: MiSeq

**References cited**

Amaral-Zettler, L.A., McCliment, P., Ducklow, H.W., Huss, S.M. (2009) A method for studying protistan diversity using massively parallel sequencing of V9 hypervariable regions of small-subunit ribosomal RNA genes. PLoS ONE 4: e6372. <https://doi.org/10.1371/journal.pone.0006372>

Blaxter, M. L., De Ley, P., Garey, J. R., Liu, L. X., Scheldeman, P., Vierstraete, A., Vanfleteren, J. R. et al. (1998) A molecular evolutionary framework for the phylum Nematoda. Nature, 392: 71–75. <http://www.nature.com/articles/32160>

Faircloth, B.C., Glenn, T.C. (2012) Not all sequence tags are created equal: designing and validating sequence identification tags robust to indels. PLoS ONE 7(8): e42543. <https://doi.org/10.1371/journal.pone.0042543>

Folmer, O., Black, M., Hoeh, W., Lutz, R., Vrijenhoek, R. (1994) DNA primers for amplification of mitochondrial cytochrome c oxidase subunit I from diverse metazoan invertebrates. Mol. Mar. Biol. Biotechnol. 3:294–99. <https://doi.org/10.1371/journal.pone.0013102>

Fonseca, V.G., Carvalho, G.R., Sung, W., Johnson, H.F., Power, D.M., Neill, S.P., Packer, M., et al. (2010) Second-generation environmental sequencing unmasks marine metazoan biodiversity. Nature Communications, 1: article 98. <https://doi.org/10.1038/ncomms1095>

Geller, J. B., Meyer, C. P., Parker, M., and Hawk, H. (2013) Redesign of PCR primers for mitochondrial cytochrome c. oxidase subunit i for marine invertebrates and application in all-taxa biotic surveys. Molec. Ecol. Res. 13, 851–861. <https://doi.org/10.1111/1755-0998.12138>

Hirai, J., Kuriyama, M., Ichikawa, T., Hidaka, K., Tsuda, A. (2015) A metagenetic approach for revealing community structure of marine planktonic copepods. Molecular Ecology Resources, 15: 68–80. <https://doi.org/10.1111/1755-0998.12294>

Leray, M., Yang, J.Y., Meyer, C.P., Mills, S.C., Agudelo, N., Ranwez, V., et al. (2013) A new versatile primer set targeting a short fragment of the mitochondrial COI region for metabarcoding metazoan diversity: Application for characterizing coral reef fish gut contents. Frontiers in Zoology 10: 34. <https://doi.org/10.1186/1742-9994-10-34>

Meyer, M., Stenzel, U., Hofreiter, M. (2008) Parallel tagged sequencing on the 454 platform. Nat Protoc 3, 267–278 <https://doi.org/10.1038/nprot.2007.520>

Rossel, S., Kaiser, P., Bode-Dalby, M., Renz, J., Laakmann, S., Auel, H., Hagen, W., Arbizu, P. M., Peters, J. (2023) Proteomic fingerprinting enables quantitative biodiversity assessments of species and ontogenetic stages in Calanus congeners (Copepoda, Crustacea) from the Arctic Ocean. Molecular Ecology Resources 23: 382–395. <https://doi.org/10.1111/1755-0998.13714>

Stoeck, T., Bass, D., Nebel, M., Christen, R., Jones, M.D.M., Breiner, H.W., Richards, T.A. (2010) Multiple marker parallel tag environmental DNA sequencing reveals a highly complex eukaryotic community in marine anoxic water. Mol. Ecol., 19:21–31. <https://doi.org/10.1111/j.1365-294X.2009.04480.x>

Wangensteen, O.S., Palacín, C., Guardiola, M., Turon, X. (2018) DNA metabarcoding of littoral hard-bottom communities: high diversity and database gaps revealed by two molecular markers. PeerJ 6: e4705. <https://doi.org/10.7717/peerj.4705>.
