# Supplementary material for: MetaZooGene Intercalibration Experiment (MZG‐ICE): Metabarcoding Marine Zooplankton Diversity of the Global Ocean
Source: Mol Ecol Resour. 2025 Dec 26;26(1):e70090. doi: 10.1111/1755-0998.70090 (PMC12742283; doi:10.1111/1755-0998.70090)
Supplement: Supplementary file 2 — Appendix S2: men70090‐sup‐0002‐AppendixS2.docx. [file MEN-26-e70090-s001.docx]

**Supplemental Information for:**

**MetaZooGene Intercalibration Experiment (MZG-ICE):**

**Metabarcoding Marine Zooplankton Diversity of the Global Ocean**

Leocadio Blanco-Bercial, Jennifer M. Questel, Paola G. Batta-Lona, Ruben Escribano, Tone Falkenhaug, Junya Hirai, Jenny A. Huggett, Pedro Martinez Arbizu, Katja T.C.A. Peijnenburg, Leonie Suter, Agata Weydmann-Zwolicka, Stacey Dubbeldam, Elza Duijm, Elizaveta Ershova-Menze, Carolina E. Gonzalez, Ashrenee Govender, Johan Groeneveld, Sahar Khodami, Ryszard Kuczyński, Anna J. MacDonald, Monika Mioduchowska, Andrea M. Polanowski, Rocio Rodriguez-Perez, Todd D. O’Brien, Ann Bucklin

**Table of Contents:**

| **Table of Contents** | Page 1 |
| --- | --- |
| **Suppl. Figure 1A.** Polar graphs for Baltic COI sequence numbers. | Page 2 |
| **Suppl. Figure 1B.** Polar graphs for BATS COI sequence numbers. | Page 3 |
| **Suppl. Figure 1C.** Polar graphs for Fjord COI sequence numbers. | Page 4 |
| **Suppl. Figure 1D.** Polar graphs for North COI sequence numbers. | Page 5 |
| **Suppl. Figure 1E.** Polar graphs for NWAtl COI sequence numbers. | Page 6 |
| **Suppl. Figure 1F.** Polar graphs for NWPac COI sequence numbers. | Page 7 |
| **Suppl. Figure 1G.** Polar graphs for SAtl COI sequence numbers. | Page 8 |
| **Suppl. Figure 1H.** Polar graphs for SEPac COI sequence numbers. | Page 9 |
| **Suppl. Figure 1I.** Polar graphs for SWInd COI sequence numbers. | Page 10 |
| **Suppl. Figure 1J.** Polar graphs for Tasman COI sequence numbers. | Page 11 |


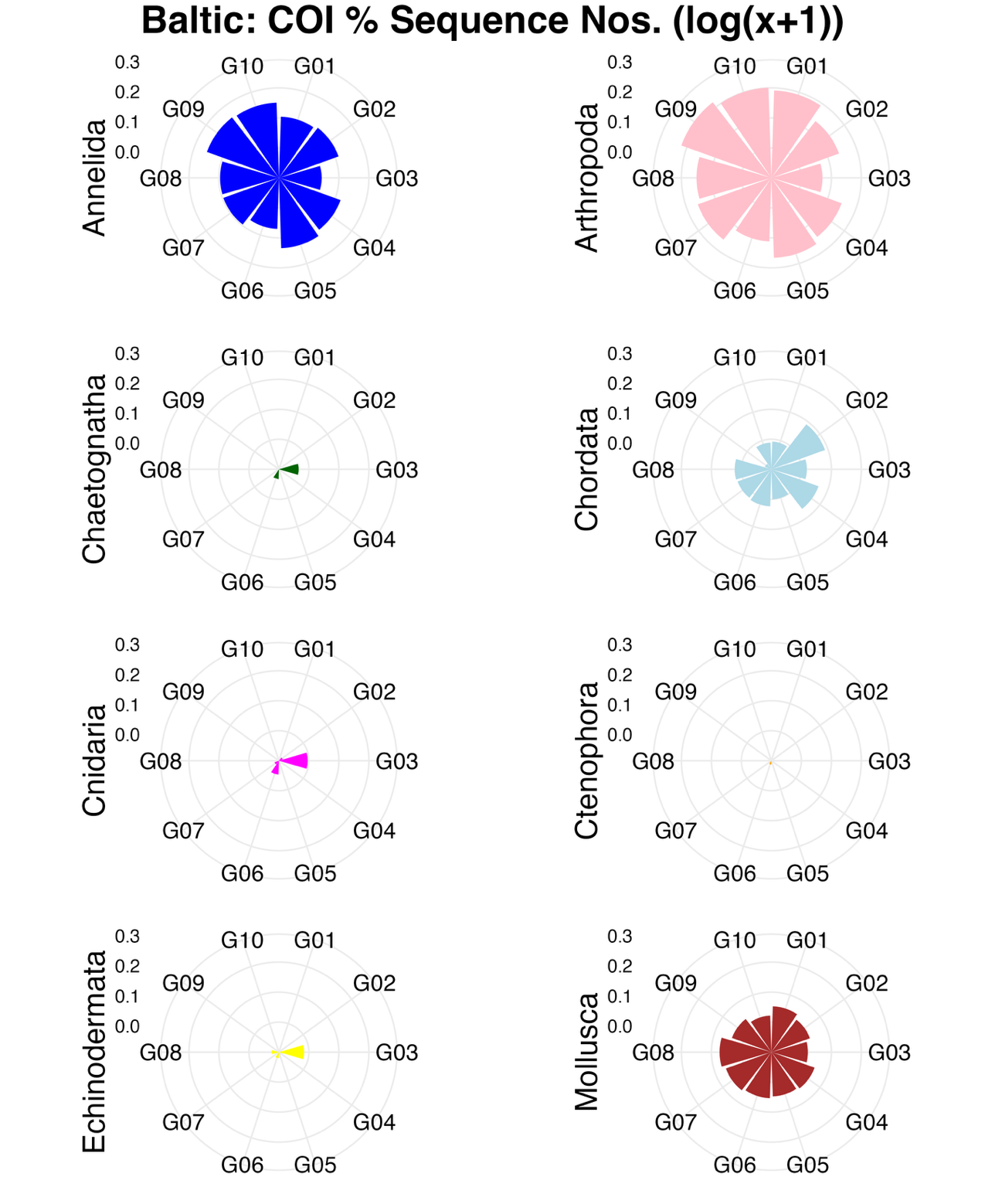


**Suppl. Figure 1A.** Polar graphs for Baltic COI sequence numbers (proportional, log(x+1)). Plots show phyla detected with highest abundances across all samples. The scaling follows the percentage scale range plotted in heatmaps. MZG-ICE group name abbreviations are explained in Figure 1.


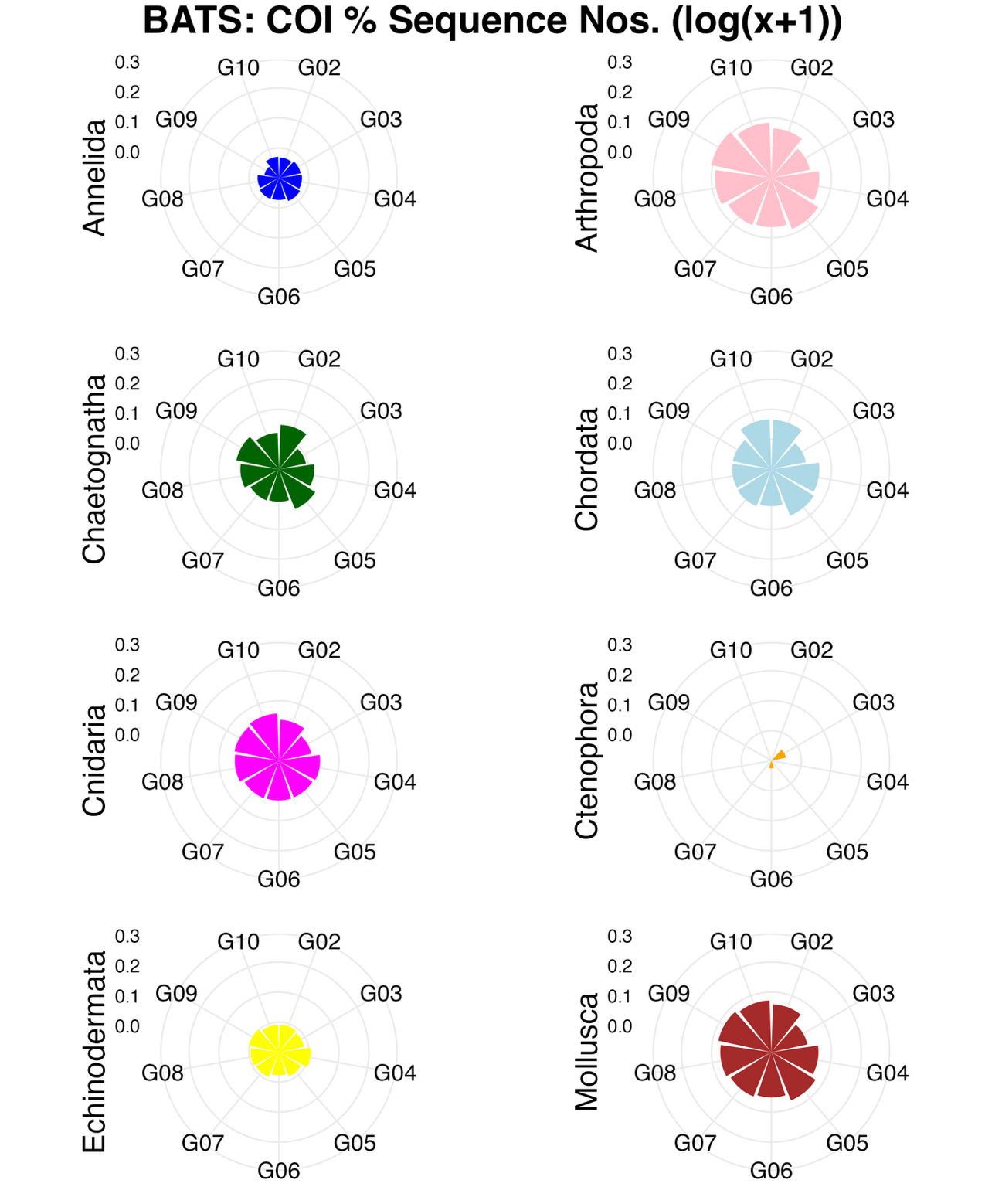


**Suppl. Figure 1B.** Polar graphs for BATS COI sequence numbers (proportional, log(x+1)).


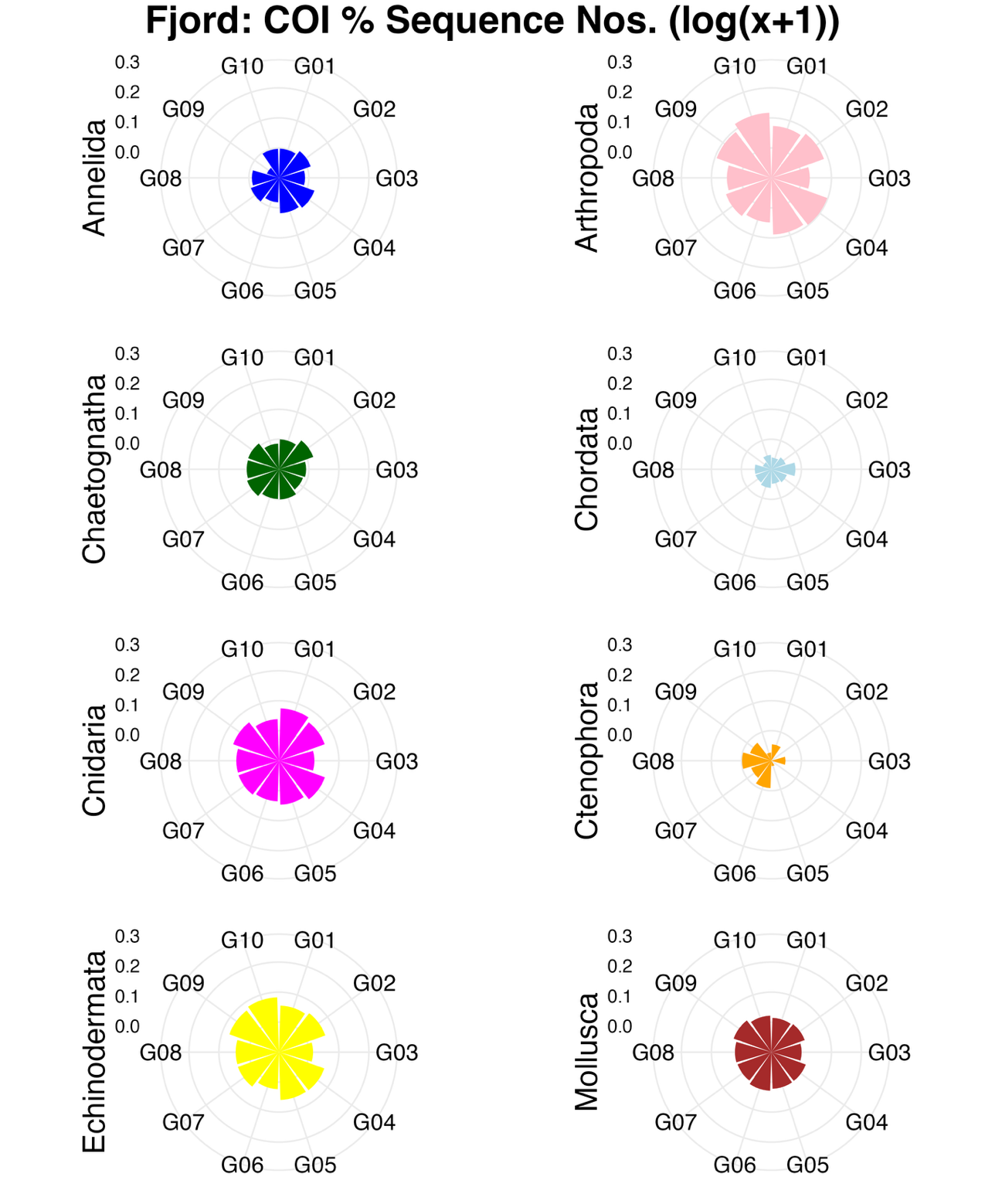


**Suppl. Figure 1C.** Polar graphs for Fjord COI sequence numbers (proportional, log(x+1)).


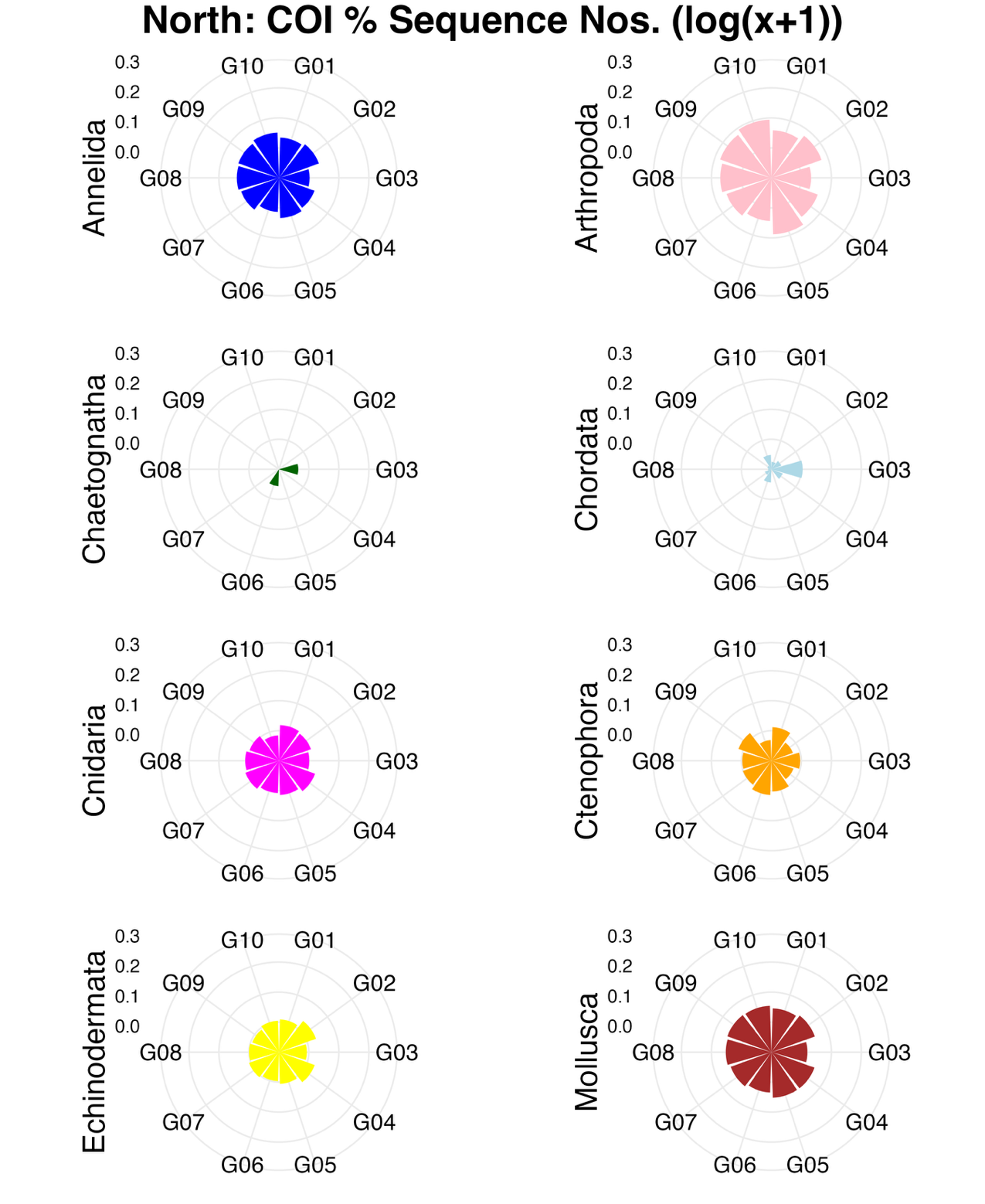


**Suppl. Figure 1D.** Polar graphs for North COI sequence numbers (proportional, log(x+1)).


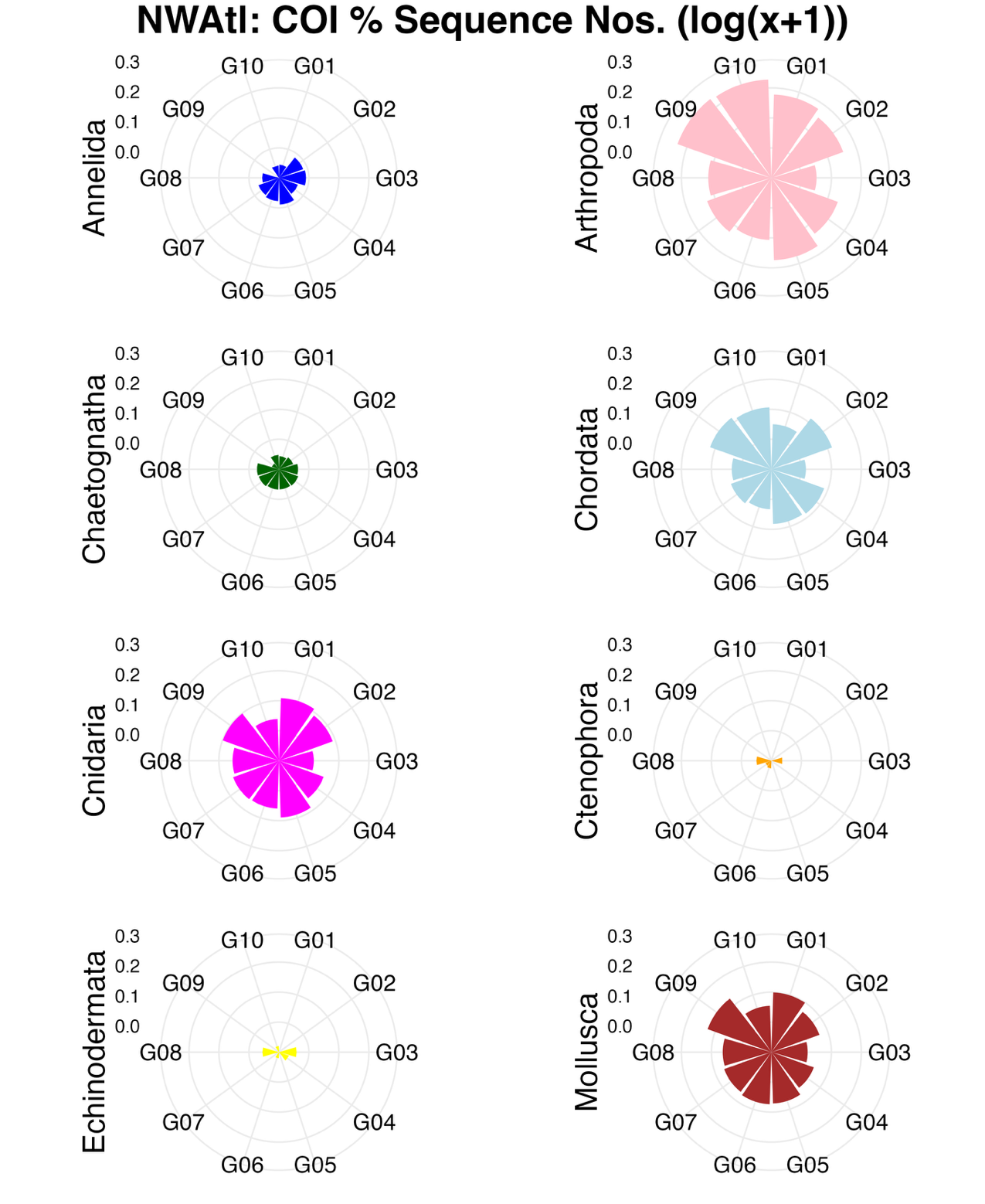


**Suppl. Figure 1E.** Polar graphs for NWAtl COI sequence numbers (proportional, log(x+1)).


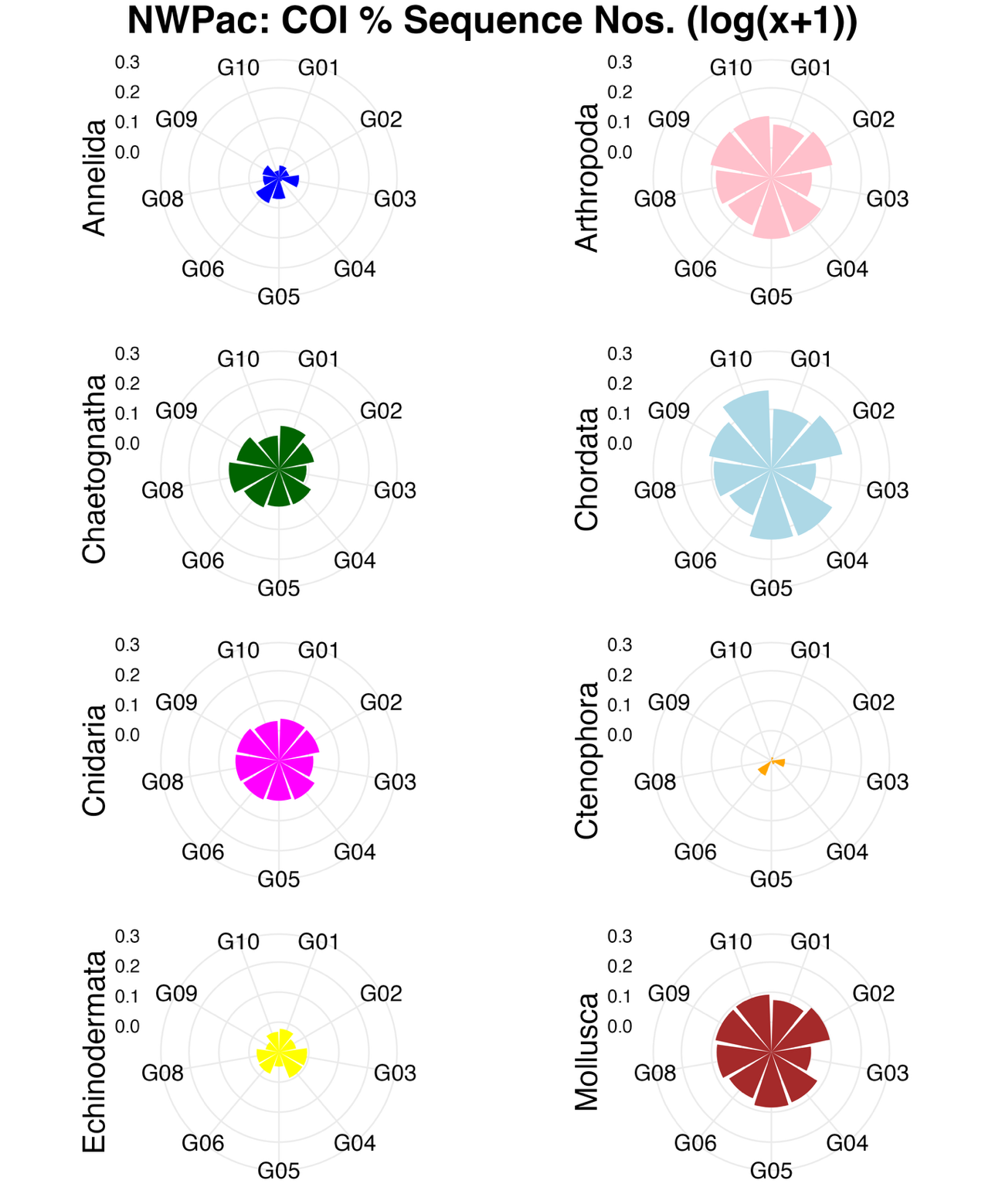


**Suppl. Figure 1F.** Polar graphs for NWPac COI sequence numbers (proportional, log(x+1)).


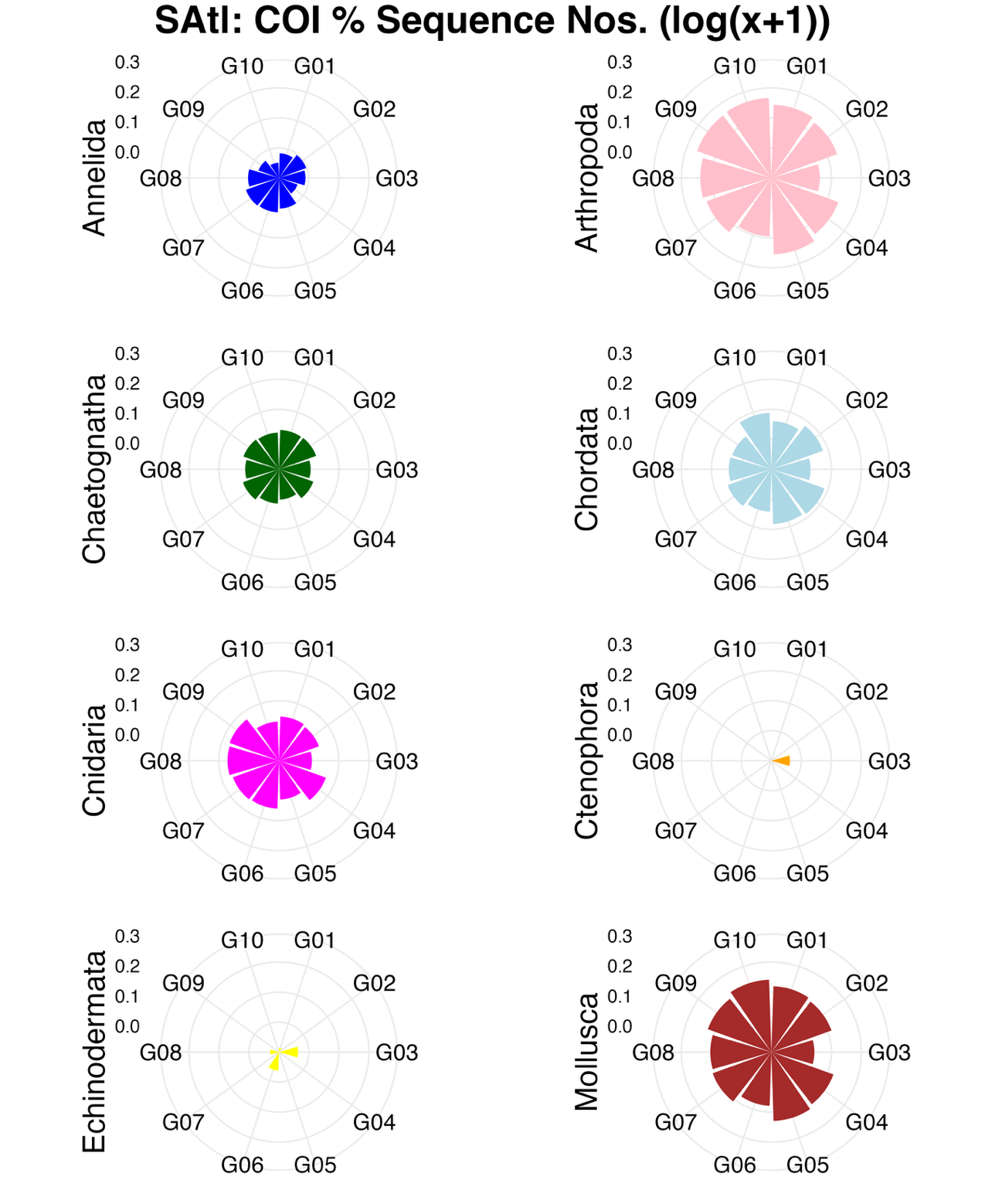


**Suppl. Figure 1G.** Polar graphs for SAtl COI sequence numbers (proportional, log(x+1)).


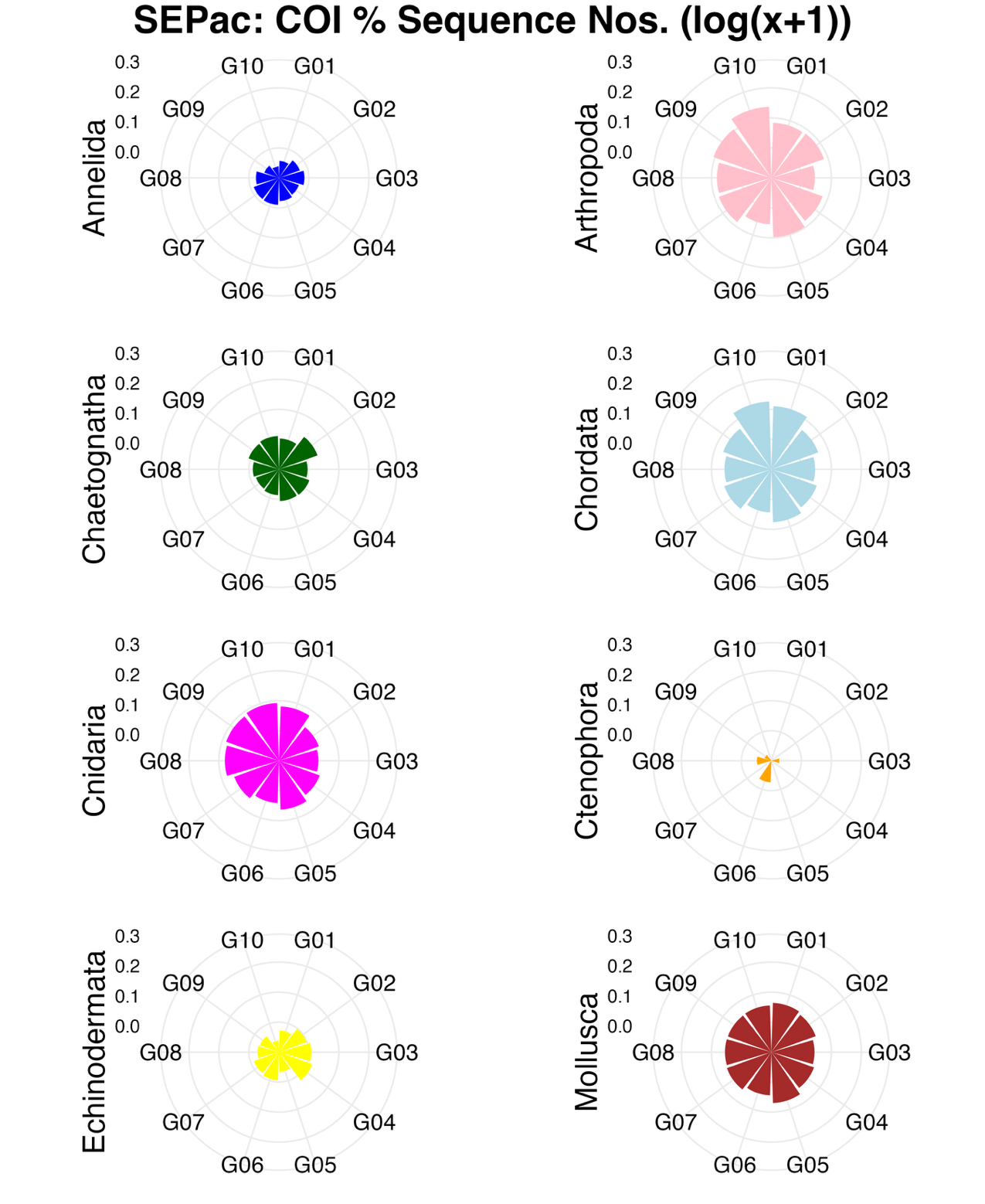


**Suppl. Figure 1H.** Polar graphs for SEPac COI sequence numbers (proportional, log(x+1)).


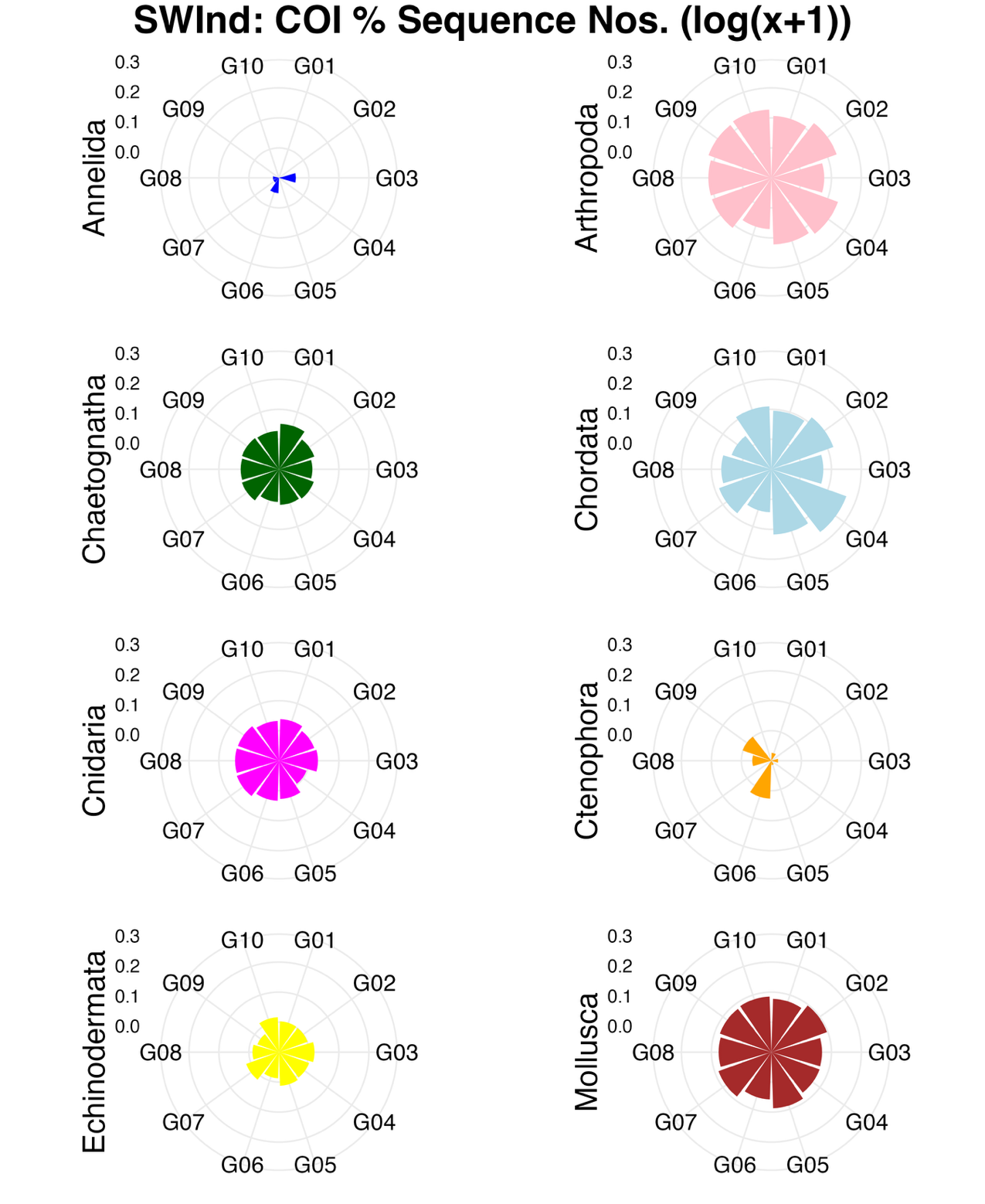


**Suppl. Figure 1I.** Polar graphs for SWInd COI sequence numbers (proportional, log(x+1)).


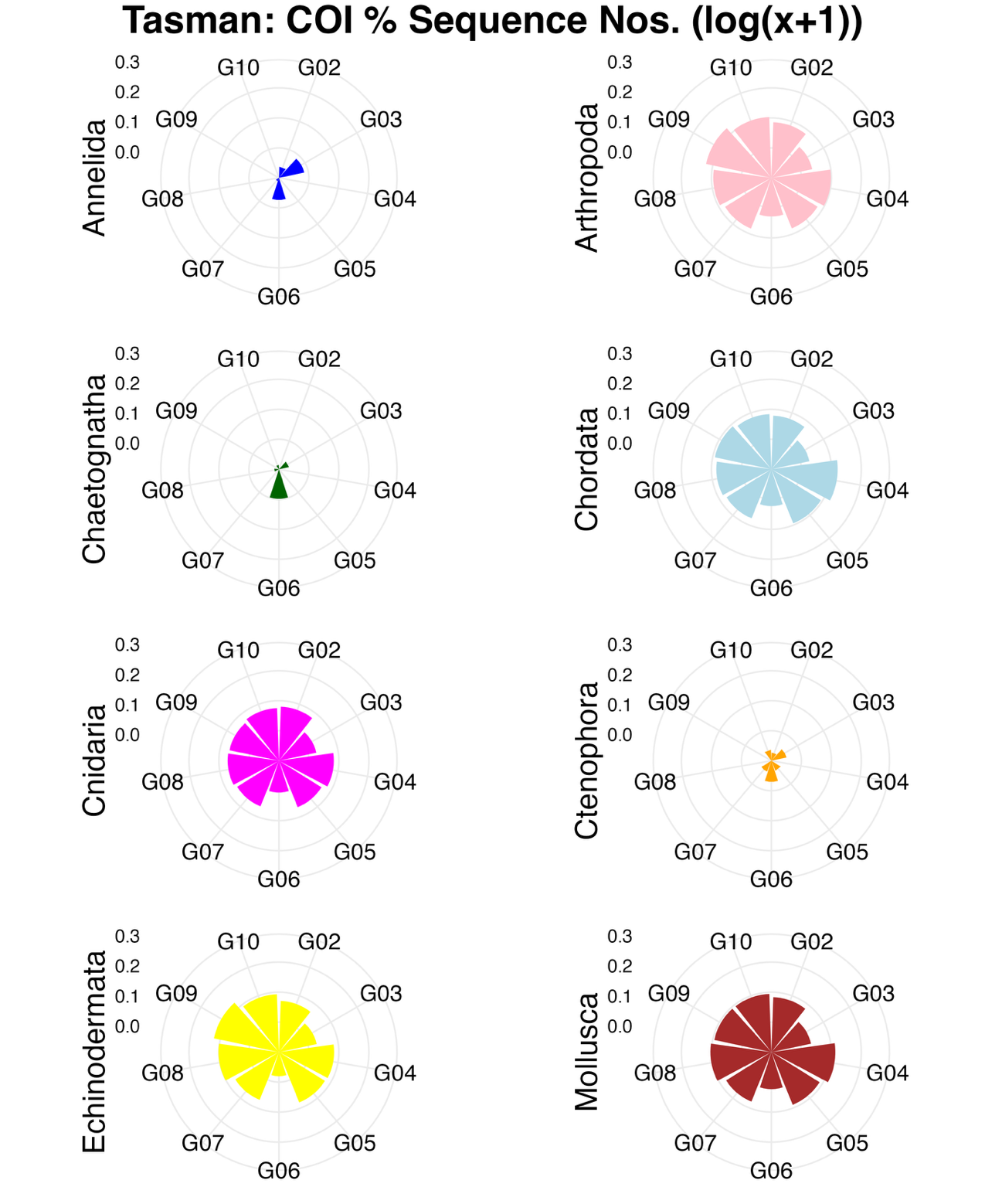


**Suppl. Figure 1J.** Polar graphs for Tasman COI sequence numbers (proportional, log(x+1)).
